# Integrin restriction by *miR‐34* protects germline progenitors from cell death during aging

**DOI:** 10.1111/acel.14131

**Published:** 2024-03-07

**Authors:** Noam Perry, Racheli Braun, Aya Ben‐Hamo‐Arad, Diana Kanaan, Tal Arad, Lilach Porat‐Kuperstein, Hila Toledano

**Affiliations:** ^1^ Department of Human Biology, Faculty of Natural Sciences University of Haifa Haifa Israel; ^2^ Biomedical Engineering Faculty Technion IITs Haifa Israel

**Keywords:** aging, *drosophila*, integrin, micro‐RNAs, *miR‐34*, phagoptosis, spermatogenesis

## Abstract

During aging, regenerative tissues must dynamically balance the two opposing processes of proliferation and cell death. While many microRNAs are differentially expressed during aging, their roles as dynamic regulators of tissue regeneration have yet to be described. We show that in the highly regenerative *Drosophila* testis, *miR‐34* levels are significantly elevated during aging. *miR‐34* modulates germ cell death and protects the progenitor germ cells from accelerated aging. However, *miR‐34* is not expressed in the progenitors themselves but rather in neighboring cyst cells that kill the progenitors. Transcriptomics followed by functional analysis revealed that during aging, *miR‐34* modifies integrin signaling by limiting the levels of the heterodimeric integrin receptor αPS2 and βPS subunits. In addition, we found that in cyst cells, this heterodimer is essential for inducing phagoptosis and degradation of the progenitor germ cells. Together, these data suggest that the *miR‐34*—integrin signaling axis acts as a sensor of progenitor germ cell death to extend progenitor functionality during aging.

## Graphical abstract

Preservation of a germline progenitor pool is essential for maintaining spermatogenesis throughout adulthood and aging. This study describes key factors of the phagoptosis pathway that regulate the rate of spontaneous germ cell death (GCD) in Drosophila to modulate spermatogenesis during aging.

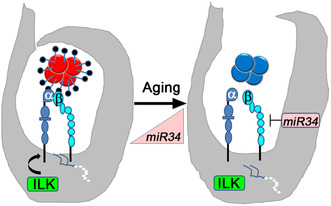

**Abbreviations**
3’UTR: 3′ untranslated region
*C. elegans*
: *caenorhabditis elegans*
CEB: cytoplasmic extraction buffer
CPM: counts per million
FC: fold Change
GCD: germ cell death
GFP: green fluorescent protein
GSCs: germline stem cells
miRNAs: microRNAs
PCD: programed cell death
RT: reverse transcription
αPS2: Inflated (If)
βPS: Myospheroid (Mys)

## 1 INTRODUCTION

The regeneration of adult tissues begins with a small population of stem cells that upon first division generate more proliferative, yet still uncommitted types of differentiated progenitor cells. Although newly formed, the progenitor cells of several adult tissues, including brain, blood and testis, undergo spontaneous programed cell death (PCD) (Alenzi et al., 2009; Allan et al., 1992; Lu et al., 2011; Rodriguez et al., 1997; Sierra et al., 2010; Yacobi‐Sharon et al., 2013). Therefore, during adulthood and aging, these progenitors must dynamically coordinate proliferation, differentiation and PCD to comply the tissue needs.

The *Drosophila* testis is a highly regenerative tissue in which spermatogenesis is initiated by unipotent germline stem cells (GSCs) that continuously divide and differentiate to generate mature sperm cells. Following asymmetric GSC division, one daughter cell remains within the niche, whereas the other, a displaced progenitor cell, mitotically transit amplifies four times with incomplete cytokinesis to yield 2–16 interconnected spermatogonia progenitor cells. These progenitors are then terminally differentiate into spermatocytes that eventually supply differentiated, short‐lived mature sperm cells. Although the testis significantly decreases in size during aging due to a reduced number of germ cells, spermatogenesis is, nonetheless, maintained until an advanced age. (Boyle et al., 2007; Cheng et al., 2008; Epstein et al., 2017; Inaba et al., 2011; Toledano et al., 2012; Wallenfang et al., 2006).

In notable resemblance to mammals, about a quarter of the newly emerging spermatogonia progenitors are spontaneously eliminated by germ cell death (GCD) (Allan et al., 1992; Lu & Yamashita, 2017; Rodriguez et al., 1997; Yacobi‐Sharon et al., 2013). The underlying mechanism of GCD is phagoptosis, a cell nonautonomous process in which neighboring cyst cells engulf live spermatogonia progenitors and degrade their contents (Zohar‐Fux et al., 2022). Knocking down components of the phagocytic machinery, such as *rab5* within cyst cells, resulted in complete inhibition of GCD (Zohar‐Fux et al., 2022).

Here, we show that during aging, GCD remains constant. This is in marked contrast to the aging‐related decline in stem cell numbers and reduction in their division rate (Boyle et al., 2007; Cheng et al., 2008; Toledano et al., 2012; Wallenfang et al., 2006), suggesting that GCD is dynamically regulated during aging. We postulated that this dynamic regulation is mediated by the function of microRNAs (miRNAs), an established class of mRNA inhibitors. Across organisms, the expression levels of selected miRNAs change with age and rather than acting in an “all‐or‐none” manner, miRNAs can tune signals strength (Garg & Cohen, 2014). We found that the evolutionarily conserved miRNA *miR‐34* (Liu et al., 2012) is expressed in phagocytic cyst cells that regulate GCD and that *miR‐34* levels increase during aging. We also found that integrin signaling regulates GCD within cyst cells. Integrin receptors correspond to heterodimeric transmembrane proteins comprising α and β subunits. In *Drosophila*, the integrin family consists of five αPS subunits (αPS1‐5) and two β subunits βν and βPS (Myospheroid or Mys), some of which are cell‐specific and can form unique α‐β heterodimer pairs (Brown et al., 2000). We found that the αPS2 (Inflated, if)‐βPS heterodimer is expressed in phagocytic cyst cells during phagoptosis and serves to regulate GCD. Finally, we found that the age‐related effect of *miR‐34* is to limit the level of integrin signaling so as to enable complete spermatogenesis throughout adulthood and aging.

## 2 RESULTS

### 2.1 GCD remains constant during aging

**FIGURE 1:**
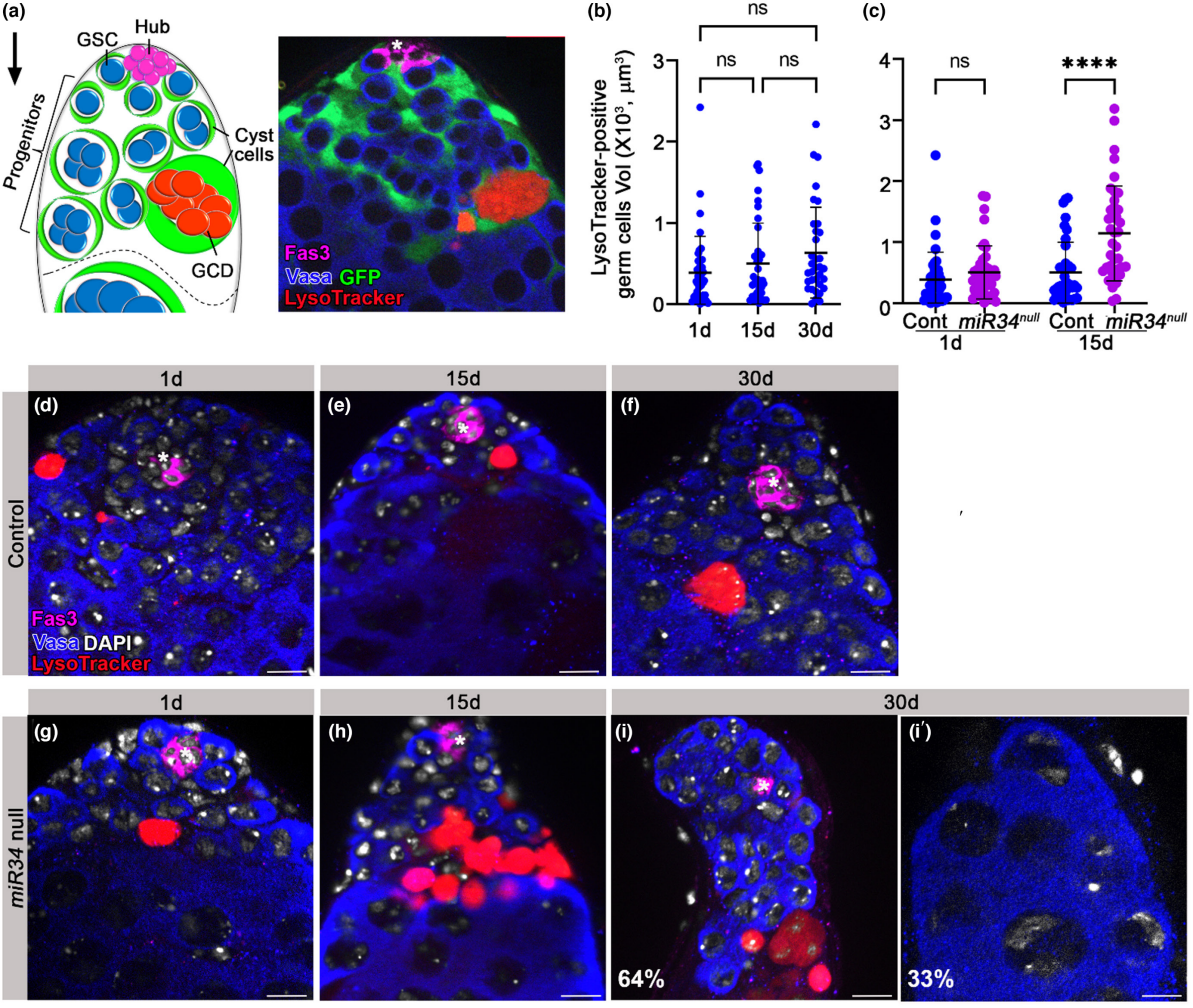
GCD increases at the apical tip of the testis of aged *miR‐34* null. (a) Schematic representation and immunofluorescent image of the apical tip of the testis (side‐view). GSCs (blue) flanked by CySCs (green) are both attached to the hub (magenta). Spermatogonia germ cells (blue) are transit‐amplified progenitor cells encapsulated by cyst cells (green). Cyst cells also induce GCD of targeted spermatogonia (red). The differentiation axis (arrow) runs from the apical (stem cell niche) to the basal end (sperm maturation). In the immunofluorescent image, the hub was labeled with anti‐Fas3 antibodies (magenta) and germ cells with anti‐Vasa (blue) antibodies. GCD was labeled with LysoTracker (red) and cyst cells were genetically labeled with GFP (green). (b, c) Quantification of the volume of LysoTracker‐positive germ cells as measured with Imaris in the testes of 1, 15 or 30 day‐old wild‐type control males (*w1118*, blue dots) and from 1 or 15 day‐old *miR‐34* null males (purple dots). Note no significant change in GCD during aging of wild‐type males (b) and age‐related increase in GCD in testes of 15 day‐old *miR‐34* null (c) Statistical significance was determined by a Kruskal‐Wallis test; ****p ≤ 0.0001 and ns = not significant. (d‐i) Representative images of the apical tip of the testes of 1, 15 or 30 day‐old wild‐type (*w1118*, d–f) or *miR‐34* null (g–i’) males. Testes were stained with LysoTracker (red, GCD), DAPI (nuclei) and immunostained for Vasa (blue, germ cells) and Fas3 (green, hub). The total number of testes scored: Control (*w1118*) 1 day‐old (*n* = 40), 15 day‐old (*n* = 39) and 30 day‐old (*n* = 34); *miR‐34* null 1 day‐old (*n* = 43) and 15 day‐old (*n* = 39). Asterisks mark the hub and scale bars represent 10 μm. Note the accelerated aging in testes of *miR‐34* null flies, the increase in GCD at 15 days and complete loss of the stem cell niche and regeneration at 33% of testes from 30‐days old males.

At the apical tip of adult *Drosophila* testes, one to several spermatogonia progenitors are spontaneously eliminated by neighboring cyst cells via GCD, as illustrated in Figure 1a. The three cell types that comprise the apical tip, namely, hub cells, cyst cells, and live and dying germ cells, are shown in an integrative fluorescent image (Figure 1a). To determine if GCD is affected by aging, testes dissected from control (*w1118*) flies at different ages were examined following staining with Vasa to mark live germ cells and with LysoTracker to mark germ cell debris (Figure 1b–f). GCD events can be identified with LysoTracker given how targeted progenitors are engulfed when still alive, with acidification persisting throughout the subsequent phagoptosis process (Kanaan et al., 2023; Yacobi‐Sharon et al., 2013; Zohar‐Fux et al., 2022). We then compared the volume of LysoTracker‐positive debris in testes from young (2 day‐old), mid‐aged (15 day‐old) and aged (30 day‐old) males (Figure 1b, d–f). Although testis regeneration significantly declines with age (Boyle et al., 2007; Cheng et al., 2008; Epstein et al., 2017), the average volume of debris remained constant in all three age groups (Figure 1b). The lack of accumulation of germ cell debris observed in the apical tip indicates that mechanisms might be in place that regulate GCD and maintain an active progenitor pool during aging.

To determine if *miR‐34*, which has previously shown to modulate neuronal loss during aging (Liu et al., 2012), plays a role in phagoptosis of spermatogonia, testes from *miR‐34* null flies were examined (Figure 1c,g–i’). Prior to examination, *miR‐34* null flies were outcrossed for five generations with controls to obtain an outcrossed homozygotic *miR‐34* null strain (Figure S1). As compared to young controls, the mean volume of LysoTracker‐positive debris in the testes of *miR‐34* null males of the same age did not change significantly, suggesting that *miR‐34* does not affect progenitor survival during development and/or early adulthood. However, as compared to mid‐aged controls, the volume of debris in the testes of mid‐aged *miR‐34* null flies increased significantly (Figure 1c–h), indicating that less progenitors are available for tissue regeneration. Finally, the testes of aged *miR‐34* null presented accelerated aging, with 67% of the testes exhibiting a narrow apical tip (Figure 1i) and 33% having lost the stem cell niche (Figure 1i’). In these samples, we found only remnants of differentiated spermatocyte germ cells without hub, stem or progenitor cells (Figure 1i’). These spermatocytes are capable of undergoing differentiation only once to produce mature sperm cells, after which regeneration is halted, ultimately resulting in an empty testis. At the same time, all testes of aged controls contained a functional niche (Figure 1f). These data suggest that *miR‐34* acts to maintain progenitor germ cells during aging, at least in part, by negatively regulating GCD.

### 2.2 *miR‐34* levels increase during aging

**FIGURE 2:**
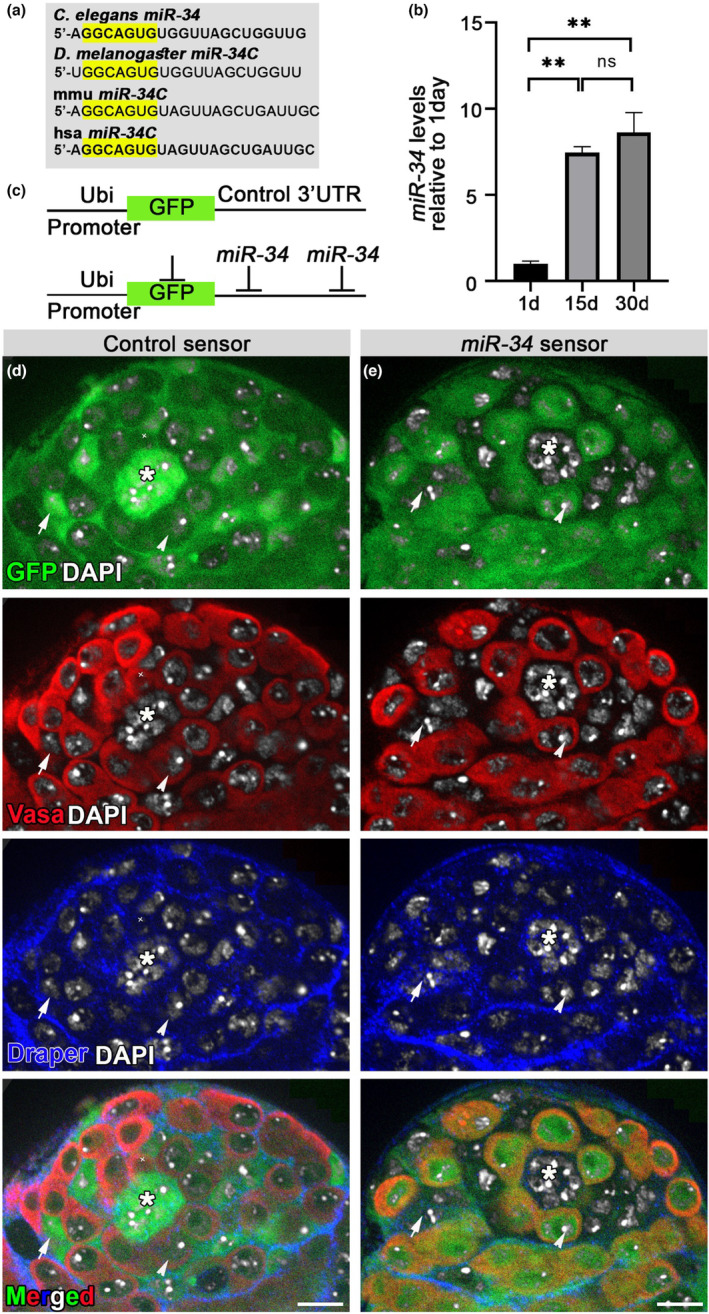
*miR‐34* is expressed in somatic cells and its levels increase during aging. (a) *miR‐34* sequence as expressed in four organisms: *C. elegans*, *Drosophila*, mouse (mmu) and humans (hsa). The evolutionarily conserved seed sequence is marked in yellow. (b) qRT‐PCR of mature *miR‐34* relative to control (*2S rRNA*) in the testes of 1, 15 or 30 day‐old wild‐type (*w1118*) males. Levels are normalized to those of 1‐day‐old adults. Error bars denote s.d. of three biological repeats, each performed in triplicate measurements. Note the 9‐fold increase of *miR‐34* levels at 30 days. Statistical significance was determined by one‐way ANOVA and posthoc analysis was performed with Tukey multi‐comparison test. **p values ≤0.005 between 15 and 1 day‐olds and between 30 and 1 day‐olds. (c) Schematic representation of GFP‐control and GFP‐*miR‐34* sensors. (d, e) Testes of GFP‐control sensor (d, green) and GFP‐*miR‐34* sensor (e, green) flies immunostained for Vasa to mark germ cells (red) and for Draper (blue) to mark the hub and cyst cell membranes and stained with DAPI to label the nuclei. A GFP‐control sensor was expressed in all cells at the apical tip of the testis, including the hub (asterisk), GSCs (arrowhead), CySCs, cyst cells (arrows) and spermatogonia. The GFP‐*miR‐34* sensor detected endogenous levels of *miR‐34* in the somatic niche (hub and CySCs) and in cyst cells (no GFP expression). Note that *miR‐34* was not expressed in germ cells (e, GFP is detected). Asterisks mark the hub and scale bars represent 10 μm.

*miR‐34* was first discovered in *Caenorhabditis elegans* (*C. elegans*) and is conserved from invertebrates to mammals, with all *miR‐34* orthologs sharing the same seven nucleotide‐long seed required for target identification (Figure 2a). The aging‐associated increase of GCD events in the testes of *miR‐34* null flies (Figure 1c) suggested dynamic expression of *miR‐34* in control flies. To assess *miR‐34* levels in testes dissected from control (*w1118*) flies at different ages, we performed qRT‐PCR to quantitate the mature form of *miR‐34*. Relative to what was measured in young (1 day‐old) males, *miR‐34* levels were increased 7‐fold in the testes of mid‐aged (15 day‐old) males and 9‐fold in aged males (30 day‐old; Figure 2b).

### 2.3 *miR‐34* is expressed in somatic cyst cells

To regulate GCD, *miR‐34* can be either expressed in germ cells targeted for cell death or in phagocytic cyst cells that engulf live germ cells. As miRNAs are not immunogenic, such that antibodies cannot be created and used to detect their expression, miRNA‐sensors were developed to monitor the expression pattern of a given miRNA in vivo (Perry et al., 2017). To identify cells that express *miR‐34* in the testis, we generated a green fluorescent protein (GFP) sensor that exploits the function of miRNAs as silencers of protein expression (Brennecke et al., 2003). The GFP*‐miR‐34* sensor comprised double repeats of the complementary sequence of *miR‐34* in an artificial 3′ untranslated region (3’UTR) inserted after a reporter *gfp* sequence. Sensor‐bearing cells that endogenously express *miR‐34* thus evoke a silencing mechanism that prevents GFP expression. We used this system to compare, the expression patterns of GFP‐control, where only the *gfp* sequence is introduced, and GFP‐*miR‐34* sensors, both driven by the *ubiquitin* promoter (Figure 2c). To determine which cells express *miR‐34*, we immunostained the testes of control and *miR‐34* sensors with antibodies against the Draper receptor that labeled the cyst and hub cell membranes and with anti‐Vasa antibodies that label the germ cell cytoplasm (Figure 2d,e). As shown in Figure 2d, GFP of the control sensor appeared throughout the apical tip of testes, with a brighter signal appearing in hub and cyst cells, which comprise the somatic niche. The strong GFP signal in the somatic niche was due to high *ubiquitin* levels in these cells (Figure 2d). In the GFP‐*miR‐34* sensor, despite the strong *ubiquitin* levels, the GFP signal was completely absent from cyst and hub cells, indicating that *miR‐34* was highly expressed in these cells. Moreover, *miR‐34* was not present in GSCs, in spermatogonia progenitor germ cells or in the entire germline lineage, as indicated by the high GFP levels observed (Figure 2e). Collectively, these findings indicate that *miR‐34* is expressed in phagocytic cyst cells that induce phagoptosis of targeted spermatogonia progenitors.

### 2.4 Identification of *miR‐34* targets in the testis

**FIGURE 3:**
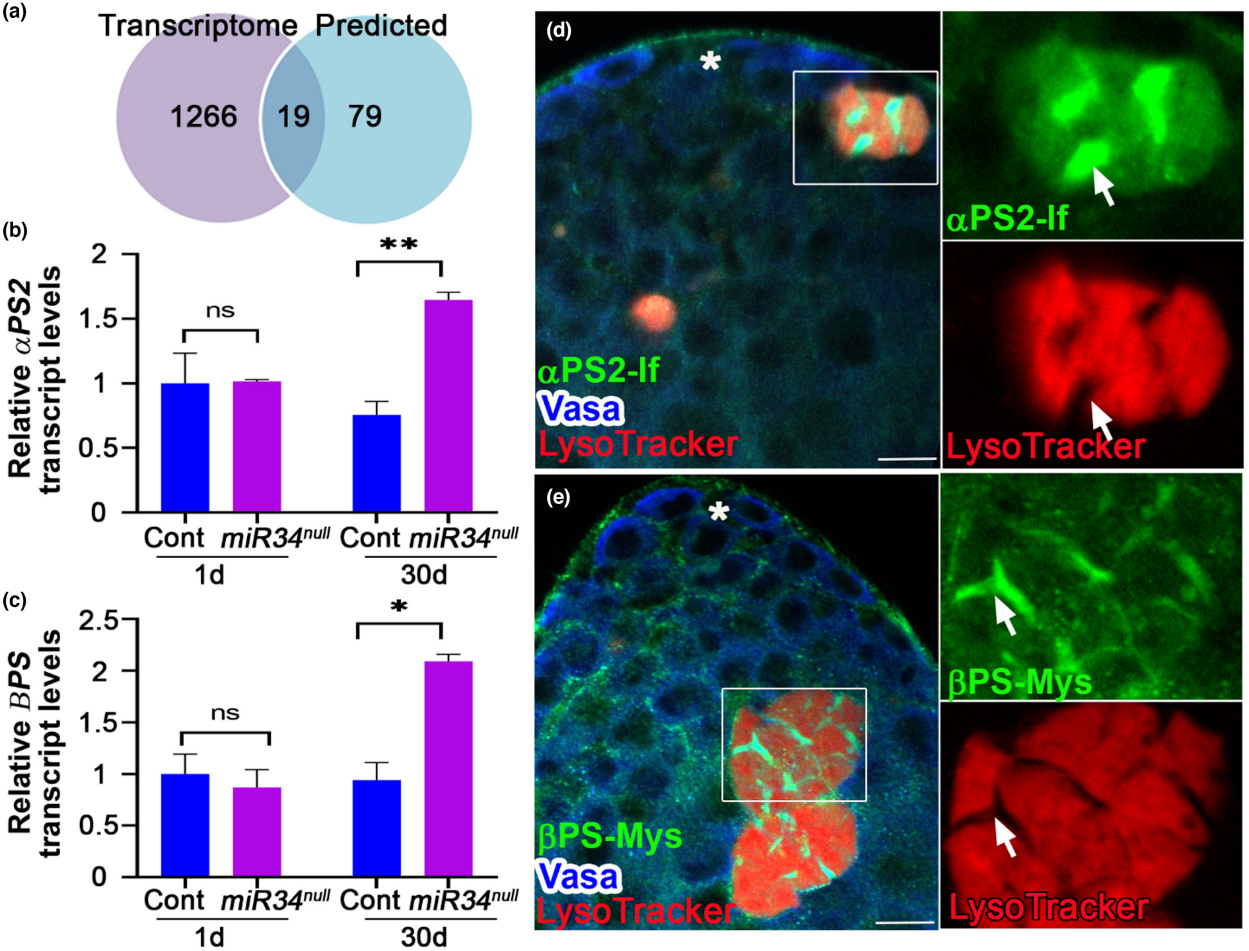
*miR‐34* regulates integrin receptor in aged testis and integrin receptor expression in GCD. (a) Venn diagram of genes increased in testis from aged (30 day‐old) *miR‐34* null mutants (purple), relative to age‐matched controls as determined by transcriptome analysis versus computationally predicted *miR‐34* targets (blue; http://www.targetscan.org/). Shown in the center of the Venn diagram are the 19 genes (Table S2) that were found in both groups; differential gene analysis (purple group) and *miR*‐*34* in silico predicted targets. (b, c) qRT‐PCR analyses of RNA of two integrins receptor subunits, *αPS2* (b) and βPS (c), extracted from testes of young and aged *miR‐34* mutants (purple), as compared to aged‐matched control (blue), relative to the average of the two normalizing genes *sdh1* and *actin42a*. Levels are normalized to those in a 1‐day old control. Note the significant 2‐fold increase in expression of the integrin receptor subunits in aged testes. Error bars denote SD of three biological repeats each performed in triplicate measurements. Statistical significance was determined as in 2d. *p* values ** ≤ 0.01 (b) or * ≤ 0.05 (c) between aged *miR‐34* mutants and controls. (d, e) Testes were stained with LysoTracker (red, GCD events) and immunostained for Vasa (blue) and αPS2 (d, green) or βPS (e, green). Note rectangles and blown‐up insets highlighting advanced GCD events. Arrows mark expression of αPS2 and βPS at the membrane of cyst cells that penetrate into notches of degraded germ cells. Asterisks mark the hub and scale bars represent 10 μm.

The age‐associated increase in *miR‐34* levels and GCD phenotype prompted us to identify the downstream targets that mediate the age‐related role of *miR‐34* in protecting the progenitor pool. miRNA‐mRNA base pairing results in translation repression and mRNA degradation (Djuranovic et al., 2012; Lau et al., 2001; Yang et al., 2013). Therefore, compared to control, the direct mRNA targets of *miR‐34* in the testis are expected to be elevated in the null mutants. To identify *miR‐34* targets in the testis, we analyzed the transcriptome of cDNA libraries (Illumina) of four RNA samples (each in four biological repeats) generated from the testis of young (1 day‐old) and aged (30 day‐old) *miR‐34* null mutants, as compared to age‐matched control (*w1118*) males. As shown in Appendix Figure S2a, clustering analysis revealed that all four biological repeats of each group clustered separately, attesting to the high quality of the samples and the ability of the analysis to identify distinct genotypes and age groups. Differential gene analysis using the edgeRclassic method yielded counts per million (CPM) and *p*‐values. After filtration based on log Fold Change (logFC ≥ 0.8), significance cutoff (*p* ≤ 0.05), and minimal CPM per each gene (≥1) determination, we obtained a group of 962 genes that showed higher expression in young *miR‐34* mutant versus controls, and 1285 genes that showed higher expression in aged mutants versus control (Figure 3a). A comparison of each of these lists of genes to the 98 in silico‐predicted *miR‐34* direct targets (Targetscan Fly; (Ruby et al., 2007)) revealed eight potential direct targets in the testes of young males (Table S1) and 19 in the testes of aged males (Table S2), four of which were increased in both young and aged mutants.

Since the phenotype of *miR‐34* null mutants is clearly related to aging, we considered the 15 candidate genes that appeared only in the aged group. Notably, these included *Eip74EF*, a previously characterized target of *miR‐34* that was shown to protect *Drosophila* brain from age‐related neurodegeneration (Liu et al., 2012), thus confirming reliability of the analysis (Table S2). The candidate genes also included those encoding two integrin receptor subunits, *αPS2* and βPS, the products of which form a heterodimer to serves to transduce integrin‐derived signals (Bokel & Brown, 2002). Transcripts of these genes each contains one recognition motif complimentary to the *miR‐34* seed sequence in its 3’UTR (Table S2). Consistent with these observations, qRT‐PCR analyses of RNA extracted from testes of young and aged *miR‐34* mutants relative to age‐matched controls showed a significant enrichment of the transcripts for the two integrin receptor subunits only in testes from aged males (Figure 3b,c). Importantly, the changes in the expression of the two integrin receptor subunits were similar in magnitude (i.e., 2‐fold increase between aged controls and *miR‐34* mutants was noted), increasing the likelihood of elevated signaling via the integrin pathway in aged *miR‐34* mutants.

### 2.5 Integrin receptors are expressed in phagocytic cyst cells

Integrin receptor subunits were previously shown to be expressed in somatic hub cells and cyst stem cells (CySCs) (Issigonis et al., 2009; Tanentzapf et al., 2007). To determine whether the αPS2 and βPS subunits are expressed in cyst cells during phagoptosis, we probed the apical tip of the testis with anti‐αPS2 and anti‐βPS antibodies (Figure 3d,e, Figure S2b,c and Movie S1). In advances stages of GCD, notches often appear in the LysoTracker staining pattern of the germ cell debris (Zohar‐Fux et al., 2022). We found that at the apical tip of the testis, the transmembrane αPS2 (Figure 3d) and βPS (Figure 3e and Movie S1) were expressed in all cyst cells and in notches where no LysoTracker staining appeared, suggesting that the membrane of the phagocytic cyst cells actively participates in the degradation process. To validate that the observed staining was not an artifact of the LysoTracker signal, we also immunostained the testis with Vasa alone to label live germ cells together with integrin receptors. With Vasa staining, GCD events appeared as empty areas (“holes”) in the stained tissues (Figure S2b‐c). Also in this staining, we found the same pattern of cyst cell membrane stained positive for βPS (Figure S2b) and αPS2 (Figure S2c) in cells undergoing GCD. It is of note that neither integrin receptor subunit was expressed in live germ cell progenitors labeled with Vasa. Together, these data show that both the αPS2 and βPS integrin receptor subunits are expressed in phagocytic cyst cells involved in phagoptosis.

### 2.6 Integrin signaling is activated in GCD

**FIGURE 4:**
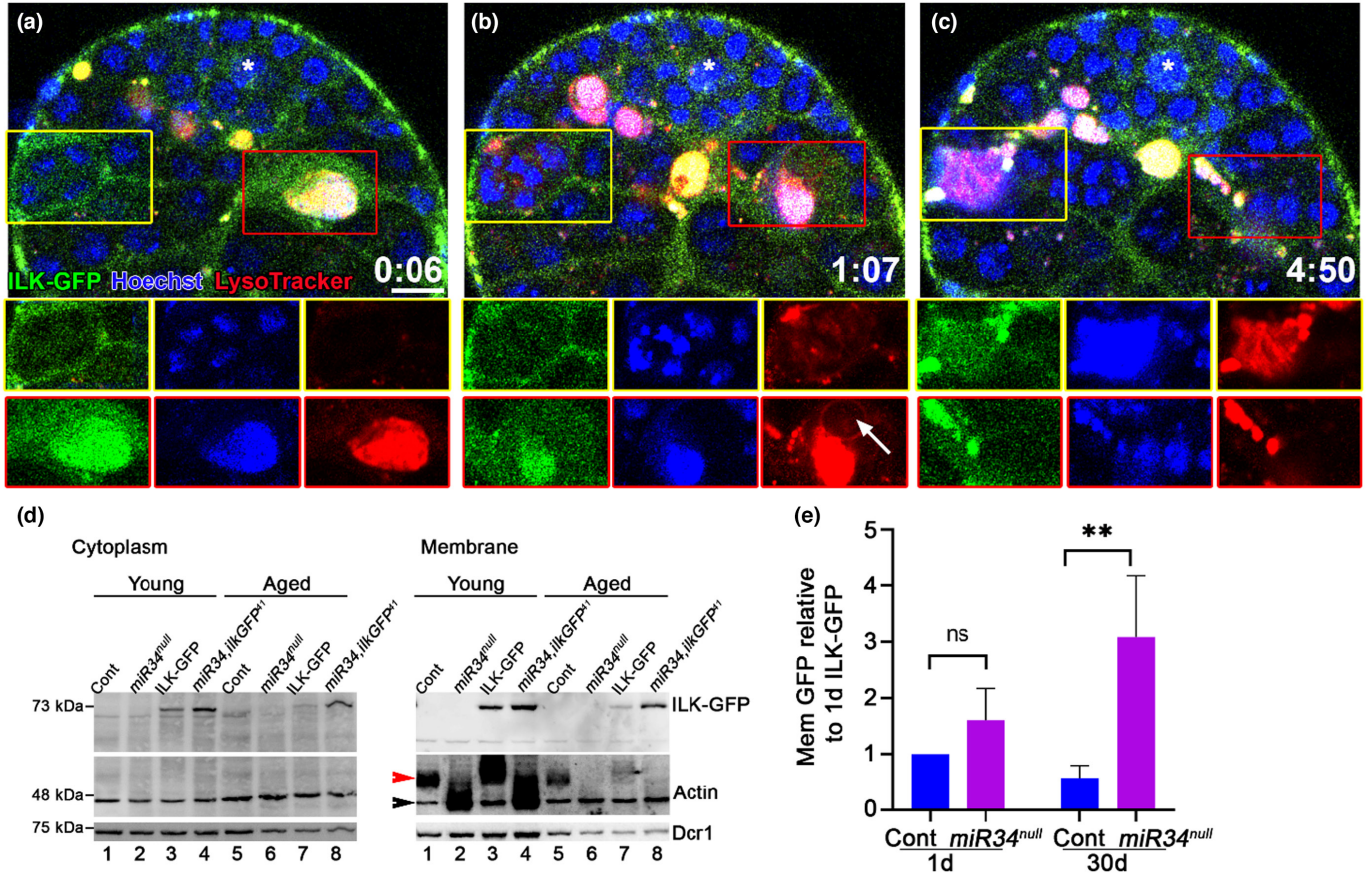
Integrin signaling is activated in GCD and increases in aged *miR‐34* null. (a–c) Snapshots of live‐imaged testis, marked with LysoTracker (red), Hoechst stain (blue, nuclei) and GFP (cyst cells, *ILK‐GFP*). Time (h:Min) is shown on the bottom left of the images. Bottom images are separate channels views of the areas surrounded by the yellow and red rectangles, highlighting dynamic expression of ILK during two GCD events. The yellow rectangle marks a new GCD event depicting packed DNA in separate nuclei (a), acidification begins after ~1 h by the onset of LysoTracker expression (b), and DNA further involuted into one bundle (c). The red rectangle marks an advanced GCD event with one bundle of DNA (a‐b) which is completely degraded within ~5 h (c). Arrow marks LysoTracker‐free blebs, which may serve as a transport mechanism to recycle components of the dying germ cells. Asterisks mark the hub and scale bars correspond to 10 μm. **(**d,e) Western blot analysis of protein extracted from cytoplasm and membrane fractions of young 1 day and aged 30 day‐old control (*w1118*), *miR‐34* null mutants, *ilk‐gfp* and *miR‐34, ilkGFP* ^*41*^ recombinant flies. (d) Shown is a representative Western blot of *n* = 4 (biological repeats) cytoplasm (left) and membrane (right) fractions of young and aged testes from control *(w1118*), *miR‐34* null, *ilk‐gfp* and *miR‐34*, *ilkGFP* ^*41*^ males, as indicated. Membranes were blotted with anti‐GFP (upper), anti‐Actin (middle) and anti‐Dicer (lower) antibodies. (e) Quantification of membrane GFP relative to Dicer; levels are normalized to ILK‐GFP levels in 1 day‐old flies (*n* = 4, biological repeats). Statistical significance was determined by one‐way ANOVA and posthoc analysis was performed with Tukey multi‐comparison test. *p* values ** ≤ 0.01 between aged control and recombinant flies. Note the higher ILK‐GFP expression in aged *miR‐34*, *ilkGFP* ^*41*^ recombinants.

Whereas the extracellular domains of integrin receptors can bind a wide variety of ligands, the cytoplasmic tails are linked to actin filaments through a network of cytoplasmic proteins, including Integrin‐Linked Kinase (ILK) (Wolfenson et al., 2013). Therefore, ILK expression and recruitment to the cell membrane is considered a reliable indicator of integrin activity (Zervas et al., 2011). To test whether ILK is present at the apical tip of the testis, we used transgenic flies expressing a protein trap of ILK‐GFP, whereby an artificial GFP‐encoding exon was inserted within the ILK‐encoding sequence (Morin et al., 2001). Here, detection of a GFP signal reports on the endogenous level and localization of ILK. Immunostaining of the testes expressing IlK‐GFP reporter with anti‐Vasa antibodies, which specifically label live germ cells, revealed that ILK is distinctly expressed in cyst cells, yet absent in germ cells (Figure S3). We then used live imaging to follow ILK‐GFP during GCD. During live imaging (*n* = 4, ~5 h each), the testes were maintained in media containing a low concentration of LysoTracker and Hoechst stain to in situ visualize changes in lysosomal activity and DNA levels, respectively (Kanaan et al., 2023). Consistent with the expression of integrin receptors, ILK‐GFP was also found in all cyst cells and was dynamically expressed in phagocytic cyst cells during GCD, suggesting that integrin signaling is activated during phagoptosis. We followed two GCD events representing distinct stages of GCD progression (Movie S2 and snapshots in Figure 4a–c). In the first event ((marked by a yellow rectangle in Figure 4)), 4‐germ cell spermatogonia, each containing packed DNA, are engulfed by a cyst cell, as seen at the beginning of the movie (Figure 4a). At this stage, no LysoTracker signal was observed, indicating that acidic lysosomes had not yet fused with the phagosome. One hour later (Figure 4b), the chromatin started to disintegrate and acidification was observed, supporting the findings of our recent publication showing that lysosomal activity occurs before DNA fragmentation (Zohar‐Fux et al., 2022). As the death process progressed (Figure 4c), the chromatin disintegrated and mixed into a single bundle. The second GCD event (marked by a red rectangle in Figure 4) had already advanced at the beginning of the movie, starting with one bundle of disintegrated chromatin (Figure 4a), which had completely degraded by the end of the movie (Figure 4c). During GCD, ILK‐GFP co‐localized with Hoechst of the engulfed spermatogonia, suggesting that the cyst cell membrane may participate in degradation (Figure 4a–c and Movie S2). ILK‐GFP was absent from the LysoTracker‐free blebs that are presumably serve as a transport mechanism to recycle components of the dying germ cells (Figure 4b). These results also highlight the gradual nature of GCD, spanning approximately 8–10 h, with the sequence of events being engulfment, acidification, and only then, DNA disintegration, and degradation.

### 2.7 Integrin signaling is hyper‐activated in aged *miR‐34* null mutants

Since *miR‐34* levels increased during aging (Figure 2d), if indeed the integrin pathway is controlled by *miR‐34*, we expected levels of the downstream ILK to be highly elevated in the older *miR‐34* null mutants. To test this hypothesis, we generated a recombinant fly combining the *miR‐34* null mutant and *ilk‐gfp* strain (hereafter, *miR‐34*, *ilkGFP* ^*41*^) and validated that this recombinant line also expressed the rescue construct containing genes that were lost during the *miR‐34* deletion process (Figure S4; (Liu et al., 2012)). To further quantify differences between control (*w1118*), *miR‐34* null, *ilk‐gfp* and *miR‐34*, *ilkGFP* ^*41*^ recombinant mutants, we extracted protein from testes dissected from young and old males of each strain. We next obtained separate membrane and cytoplasmic fractions and probed each fraction for the presence of ILK‐GFP. Western blot analysis using anti‐GFP antibodies showed similar observations in the cytoplasm and membrane fractions (Figure 4d). As expected, young control and *miR‐34* null samples contained no ILK‐GFP (Figure 4d, lanes 1 and 2 of both the cytoplasm and membrane panels). In control, where *miR‐34* is present, ILK‐GFP levels were reduced during aging (Figure 4d, lanes 3 and 7 of both the cytoplasm and membrane panels). No significant differences were observed in ILK‐GFP expression among young *ilk‐gfp* and *miR‐34*, *ilkGFP* ^*41*^ null files (Figure 4d, lanes 3 and 4 of both the cytoplasm and membrane panels). In contrast, while ILK‐GFP levels were significantly decreased in control aged flies, the aged *miR‐34*, *ilkGFP* ^*41*^ mutants showed increased expression (Figure 4d, lanes 7 and 8 of both the cytoplasm and membrane, and Figure 4e). Since elevated integrin signaling was seen in aged *miR‐34*, *ilkGFP* ^*41*^ mutants, we concluded that one of the roles of *miR‐34* is to restrict integrin signaling during aging.

As the activation of integrin anchors the actin cytoskeleton to the cell membrane (Zaidel‐Bar et al., 2007), we probed the cytoplasmic and membrane fractions by Western blotting with anti‐actin antibodies. In control samples extracted from the membrane fractions (Figure 4d, lanes 1, 3, 5, and 7), in addition to the expected 42 kDa band (black arrowhead), actin also appeared as part of a high molecular complex (red arrowhead). In contrast, in the testes of young and aged *miR‐34* null flies (Figure 4d, lanes 2, 4, 6, and 8), actin was present mostly at the 42 kDa MW. The fact that these differences in the migration of actin only appeared in the membrane compartment further support the claim that *miR‐34* affects integrin signaling.

### 2.8 Reduced βPS levels in cyst cells nonautonomously decrease GCD

**FIGURE 5:**
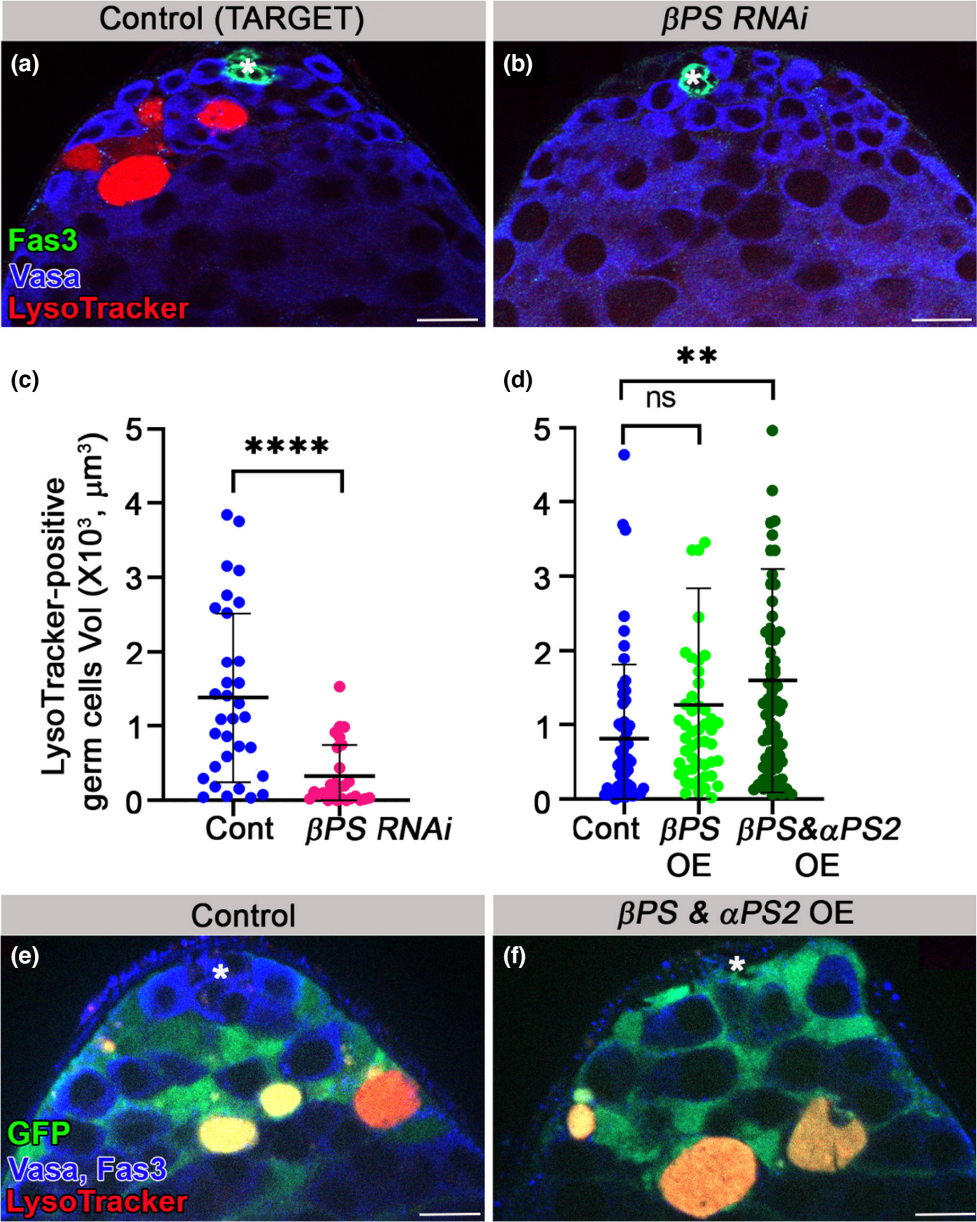
Integrin signaling by cyst cells regulates GCD. (a–c) Immunofluorescent images of testes from 7 day‐old control TARGET flies (a) *c587Gal4;Gal80ts* outcrossed to *w1118* (*n* = 32) and βPS RNAi transgene expressed in cyst cells of adult males by TARGET driver (b) *c587Gal4;Gal80ts*, *UAS‐mysRNAi* (*n* = 27). Testes were labeled with LysoTracker (red, dying germ cells) and for Fas3 (green, hub) and Vasa (blue, live germ cells). Quantification of the volume of LysoTracker‐positive germ cells as measured with Imaris (c) control, blue dots and *mys* RNAi, pink dots. Note the significant reduction in GCD in testes of βPS RNAi‐expressing flies. Statistical significance was determined by a Mann–Whitney test, ****p ≤ 0.0001. (d, e) Quantification of the volume of LysoTracker‐positive germ cells, as measured with Imaris (d) of control (*c587Gal4*; *UAS‐cytGFP/+*; blue dots), βPS OE (*c587Gal4*; *UAS‐cytGFP/UAS‐*βps*‐GFP*; light green dots) and βPS & *αPS2* OE (*c587Gal4*; *UAS‐cytGFP/UAS‐*βps, *UAS‐ αps2*; dark green dots). Note the significant increase in GCD only in testes expressing the two integrin receptor subunits in cyst cells. Immunofluorescent images of testes from control (e) and from βPS & *αPS2* overexpressing flies (F). Statistical significance was determined by a Kruskal‐Wallis test. **p ≤ 0.01; ns, not significant. Asterisks mark the hub and scale bars correspond to 10 μm.

Integrins are well‐established engulfment receptors known to mediate the phagocytosis of cellular debris by professional phagocytes (Boada‐Romero et al., 2020; Penberthy & Ravichandran, 2016). Integrins were also shown to promote phagoptosis of live neurons cocultured with activated microglia (Hornik et al., 2016). In vivo, in *Drosophila* ovary, follicle cells, which are non‐professional phagocytes, also utilize the integrin αPS3/βPS heterodimer for germline cell clearance (Serizier & McCall, 2017). Hence, to assess the contribution of cyst cell–derived integrin to GCD, a temporal and regional gene expression targeting (TARGET) system was used to induce RNAi‐mediated knockdown of βPS specifically in the cyst cells of adult flies (McGuire et al., 2004). Immunofluorescent staining with anti‐Vasa antibodies and LysoTracker staining of testes from flies raised for 7 days at the restrictive temperature of 29°C revealed that RNAi‐mediated knockdown of βPS markedly reduced GCD (Figure 5a–c). Quantification revealed a 5.6‐fold decrease in the volume of LysoTracker‐positive germ cells (Figure 5c), indicating that integrin receptors in cyst cells nonautonomously regulate GCD.

### 2.9 Co‐expression of αPS2 and βPS in cyst cells increases GCD

We next considered whether overexpression of the *αPS2* and βPS integrin receptor subunits in cyst cells was sufficient to increase germ cell phagoptosis. We found that, as compared to control, expression of βPS alone was not sufficient to affect GCD (Figure 5d). However, co‐expression of the two subunits in the cyst cells nonautonomously increased GCD, further supporting the requirement of the αPS2/βPS heterodimer for the phagoptotic process (Figure 5d–f). To assess whether integrin overexpression in cyst cells was adequate to replicate the observed stem cell niche loss in 33% of aged *miR‐34* null flies (Figure 1i’), we subjected the integrin overexpressing flies to a 30‐day aging process. Relative to control testes (*n* = 31), which all contained a functional niche, overexpression of the ßPS subunit alone resulted in a 6% loss (*n* = 34), while co‐expression of both integrin *αPS2* and ßPS subunits resulted in a 13% loss (*n* = 30) of the hub (Figure S5). These results, partially recapitulating the hub demise phenotype observed in aged *miR‐34* null flies, point to the involvement of additional targets that potentially regulate hub maintenance in aged flies.

### 2.10 Ectopic expression of MFG‐E8 mediates GCD

**FIGURE 6:**
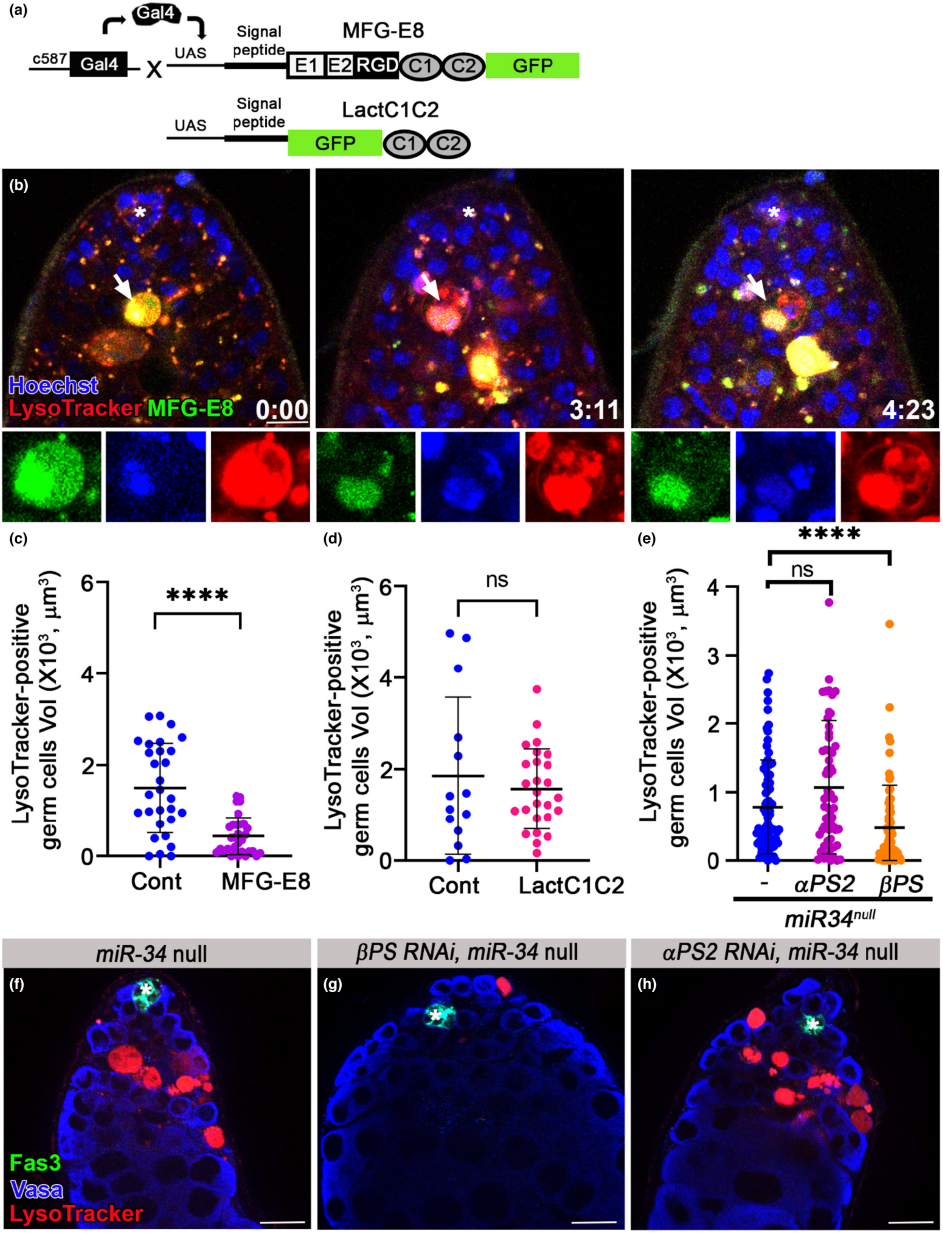
Integrin signaling by cyst cells regulates GCD. (a) Schematic representation of the secreted PS‐integrin binding protein, MFG‐E8‐GFP. MFG‐E8 includes C1 and C2 domains that bind PS and the RGD motif that binds the integrin receptor. LactC1C2‐GFP is a truncated version of MFG‐E8‐GFP that lacks the integrin‐interacting RGD motif. (b) Snapshots of live‐imaged testis, marked with LysoTracker (red), Hoechst (blue, nuclei) and MFG‐E8‐GFP (green) expressed in and secreted from cyst cells (*c587Gal4;UAS‐mfg‐e8‐gfp*). Lower panels are high‐magnification views of one GCD event (white arrow) highlighting the MFG‐E8‐mediated signal of PS exposure accumulating for ~4 h. Time (h:min) is shown on the bottom right of the images, asterisks mark the hub and scale bars correspond to 10 μm. (c–e) Quantification of the volume of LysoTracker‐positive germ cells, as measured with Imaris. (c) Control, blue dots (*C587Gal4* outcrossed to *W1118*; *n* = 29) and MFG‐E8‐GFP, purple dots (*c587gal4::uas‐mfg‐e8‐gff*; *n* = 32). Note the significant reduction in GCD in testes of MFG‐E8‐GFP‐expressing flies. (d) Control, blue dots (*C587Gal4; Gal80* ^*ts*^ outcrossed to *W1118*; *n* = 14), and LactC1C2‐GFP (*c587gal4; gal80* ^*ts*^::*uas‐lactc1c2‐gfp; n* = 26). Note that the integrin‐interacting motif is required to reduce GCD. (e) *miR‐34* null (blue dots), *αPS2* RNAi (pink dots) and βPS RNAi (orange dots) expressed in cyst cells of *miR‐34* null flies. Statistical significance was determined by a Mann–Whitney test; ****p ≤ 0.0001 and ns = not significant. Note that reduction of βPS in cyst cells is sufficient to rescue the excessive GCD in testes of *miR‐34* null. (f–h) Immunofluorescent images of testes from (f) *miR‐34* null (*tjGal4; miR‐34* null; *n* = 49), (g) βPS and *αPS2* (h) RNAi transgenes expressed in cyst cells of *miR‐34* null (*tjGal4; UAS‐mysRNAi miR‐34* null; *n* = 49), Testes were labeled with LysoTracker (red, dying germ cells) and immunostained for Fas3 (green, hub) and Vasa (blue, live germ cells). Asterisks mark the hub and scale bars correspond to 10 μm.

The most common “eat‐me” signal for phagocytosis is phosphatidylserine (PS) exposed on the cell surface of dying/dead cells. We recently showed that PS was detectable on targeted germ cell surfaces only after signs of lysosomal activity, implying that in GCD, PS does not function as the classic “eat‐me” signal that induces phagoptosis by cyst cells (Zohar‐Fux et al., 2022). In mammals, integrin recognition of PS is indirect, relying on the adaptor protein, milk fat globule‐EGF factor 8 (MFG‐E8 or Lactadherin) (Hanayama et al., 2002). MFG‐E8 is a secreted protein and includes C1 and C2 domains that bind PS and the RGD motif that binds the integrin receptor, thus bridging cell debris and phagocytes (a schematic illustration is provided in Figure 6a). To determine the function of the integrin receptor in GCD, we ectopically expressed GFP‐tagged mammalian MFG‐E8 in cyst cells (*UASmfg‐e8‐GFP*) (Tung et al., 2013). Although there is no known fly orthologue of MFG‐E8, we used the mammalian molecule to determine whether integrin regulates germ cell engulfment or degradation. Live imaging revealed a dynamic accumulation of MFG‐E8‐GFP in GCD that was maintained for a few hours, suggesting that such accumulation may facilitate degradation of engulfed spermatogonia (Figure 6b). If integrin were to only mediate engulfment, then one would expect more debris to accumulate in MFG‐E8‐expressing testes, as compared with controls. However, if integrin also mediates degradation of already engulfed contents, MFG‐E8 would be expected to accelerate this process, resulting in less LysoTracker‐positive debris. Indeed, when compared to controls, the mean volume of LysoTracker‐positive debris in the testes of MFG‐E8‐expressing males was significantly reduced (Figure 6c). To further explore if integrin recruitment facilitated engulfment and degradation, we ectopically expressed GFP‐Lact, a truncated version of MFG‐E8 that includes the C1 and C2 domains that specifically bind PS but lacks the integrin receptor interacting motif (Figure 6a) (Sapar et al., 2018). Unlike full‐length MFG‐E8, the mean volume of LysoTracker‐positive debris in the testes of GFP‐Lact‐expressing flies did not change significantly (Figure 6c,d), indicating that MFG‐E8 can facilitate GCD only when it interacts with integrin. Collectively, these findings indicate that integrin receptors in cyst cells mediate engulfment and degradation of engulfed germ cell contents.

### 2.11 Reduced βPS integrin levels prevent the accelerated GCD seen in miR‐34 null mutants

Since *miR‐34* potentially regulates several direct and indirect targets in cyst cells, we tested whether regulation of integrin signaling alone is sufficient to prevent the accelerated removal of germ cell progenitors. For this, RNAi‐mediated knockdown reduced the levels of βPS or *αPS2* in cyst cells of mid‐aged (15 day‐old) *miR‐34* null males. This was performed using the UAS‐GAL4 system to drive βPS or *αPS2* RNAi expression in cyst cells of the entire testis of *miR‐34* null flies (Figure 6e–h). As compared to *miR‐34* null males, reducing the levels of the βPS integrin receptor subunit in the cyst cells of *miR‐34* nulls was sufficient to block acceleration of GCD (Figure 6e–g). However, the reduction in *αPS2* levels did not impact GCD in the testes of *miR‐34* null flies (Figure 6e, f, h), indicating that additional αPS subunits might redundantly promote GCD. Our transcriptome analysis revealed that among the αPS1‐5 subunits found in *Drosophila*, two additional subunits, namely, *αPS1* (*mew*) and *αPS3* (*scb*), are expressed in the testis. Moreover, akin to *αPS2*, both *αPS1* and *αPS3* exhibit significantly increased expression in the testes of aged *miR‐34* null flies, as compared to controls. While *αPS1* and *αPS3* do not harbor a conserved *miR‐34* regulation site in their 3’UTR, *αPS3* does contain a poorly conserved such site in its open reading frame, suggesting that *αPS1* is a potential indirect target, and that *αPS3* could be either a direct or indirect target of *miR‐34* (Table S3). Together, these findings establish the *miR‐34*‐integrin axis as a central regulator of GCD during aging.

## 3 DISCUSSION

Our results suggest that *miR‐34* levels significantly increase in the testis during aging in order to protect progenitor cells from accelerated GCD and spermatogenesis exhaustion. We and others have shown that aging results in significantly smaller testis with reduced stem cell numbers and division rate (Boyle et al., 2007; Cheng et al., 2008; Epstein et al., 2017). However, although spermatogenesis decreases, sperm production remains throughout adulthood and aging, indicating that there are mechanisms that dynamically counter‐balance age‐related changes. Here, we showed that *miR‐34* plays an age‐related counter‐balance role to limit the amount of germline progenitors destined for GCD. GCD is a spontaneous and large‐scale process that eliminates a quarter of newly formed progenitors (Yacobi‐Sharon et al., 2013). As such, its proper restrain during aging is necessary to maintain spermatogenesis until an advanced age. In aged males, the volume of debris increased significantly in *miR‐34* null testes, as compared with controls, in which the average volume of debris remained constant in all age groups tested. This suggests that more spermatogonia progenitors are targeted for GCD or that debris lingers for a longer time and that the rate of degradation in the testes of *miR‐34* null flies is significantly slower than in controls. The underlying mechanism of GCD is nonautonomous phagoptosis, whereby cyst cells use their phagocytic machinery to engulf and degrade live germline progenitors. Reducing the levels of phagocytic factors, like *rab‐5*, within cyst cells is sufficient to block GCD (Zohar‐Fux et al., 2022). Therefore, it was not surprising that sensor analysis revealed that *miR‐34* was not expressed in the germline lineage but was strongly expressed in the somatic cyst cells that nonautonomously kill germ cell progenitors.

Several lines of evidence support our conclusion that *miR‐34* regulates integrin signaling. First, a *miR‐34* binding consensus site which serves as a translation inhibition and mRNA destabilization element is located at the 3’UTR of *αPS*2 and βPS mRNA, encoding two subunits of the heterodimer integrin receptor. Transcriptome and qRT‐PCR analysis of aged tissues revealed an increase in the mRNA levels of each of these subunits in *miR‐34* null flies, as expected from the lack of their negative regulator. Moreover, in *miR‐34* null flies, we found an increase in ILK levels and changes in membrane‐anchored actin, both of which are downstream effectors of integrin signaling activation, indicating that the increased mRNA levels of the integrin receptor subunits resulted in elevated signaling. Importantly, a reduction in the levels of the βPS subunit in *miR‐34* null males was sufficient to rescue the age‐related phenotype of accelerated GCD. Therefore, we propose that limiting integrin levels during aging is a main role of *miR‐34*, designed to prevent progenitor exhaustion. In support of the role of *miR‐34* as a negative regulator of integrin signaling are the findings that *miR‐34* represses the levels of *pat‐3*, encoding the sole β‐integrin receptor in *C. elegans* to modulate the integrin signaling involved in distal tip cell migration (Burke et al., 2015). This suggests that the *miR‐34*‐integrin axis is evolutionarily conserved.

In our study, we showed that *miR‐34*, integrin, ILK and actin are all found in somatic cells that nonautonomously regulate GCD. We also showed that in the absence of *miR‐34*, the composition of membrane‐anchored actin changed from a high molecular weight complex to the monomeric form, suggestive of force‐induced changes in the integrin–actin cytoskeleton linkage (Janostiak et al., 2014). However, it remains to be determined whether these changes are related to GCD or to other aspects of integrin signaling in the testis, such as providing mechanical force and signal transduction (Maartens & Brown, 2015). We propose that during aging, the levels of *miR‐34* increase in order to tone‐down integrin activity and thereby restrain GCD. We previously showed that the phagocytic receptor Draper is involved in engulfment of live progenitors (Zohar‐Fux et al., 2022). Here, we showed that the integrin receptor takes an active part in engulfment and degradation of already engulfed germ cells. Nonetheless, we cannot rule out the possibility that integrin signaling, as detected in testis of aged *miR‐34* null males, can also affect the chemical signals moving between the soma and the germline by transcription and/or cross‐talk with additional signaling cascades (Trappmann et al., 2012).

Finally, our results are in line with the anti‐aging role of *miR‐34* in *Drosophila* brain (Kennerdell et al., 2018; Liu et al., 2012), where *miR‐34* was found to play a neuroprotective role by targeting the pro‐apoptotic *Eip74EF*. Interestingly, *Eip74EF* was also identified in the unbiased transcriptome analysis reported here. Our sensor analysis revealed that in addition to cyst cells, *miR‐34* is also expressed in the hub, which functions as a major component of the stem cell niche. Hub cells are explicitly resilient to apoptosis (Volin et al., 2018). Therefore, it will be interesting to explore whether the *miR‐34*‐*Eip74EF* axis promotes hub protection from apoptosis. The neuroprotective role of *miR‐34* is also manifested by it repressing mRNAs of the polycomb repressive complex 2 (PRC2), thus relieving gene silencing (Kennerdell et al., 2018). *miR‐34* was first discovered in *C. elegans* and is conserved from invertebrates to mammals. Interestingly, human, mouse and fly orthologs share the same seven nucleotide‐long seed required for the target identification needed to mediate the function of *miR‐34* as an mRNA translation inhibitor (Lau et al., 2001; Yang et al., 2013). In mammals, *miR‐34* is the most prevalent miRNA target of p53, thus affecting the tumor suppressive role of p53. Moreover, *miR‐34* is often silenced in a variety of tumors, with its restored expression repressing tumor growth (Agostini & Knight, 2014; He et al., 2007). As such, targeting signaling pathways at the RNA levels with *miR‐34* may prove to be a prevalent theme in ensuring robustness in aged tissues and protection from tumorigenesis.

## 4 MATERIALS AND METHODS

### 4.1 Experimental model

Flies were raised at 25°C on freshly prepared standard cornmeal molasses agar medium. Young flies were selected upon hatching and dissected within the next 3 days of their life. Young flies designated for aging were placed in small vials (35 mL), with a maximum of 20 files (males and females) being raised. The vials were flipped every 2 days thereafter to prevent a second generation from hatching and adult fly loss. Middle‐aged flies were dissected at 15‐days and aged flies at 30‐ days. Control and manipulated flies were aged and tested at the same time. Crosses with the Gal4/UAS system were set up and maintained at 25°C. Crosses for the inducible Gal4/Gal80/UAS TARGET system (McGuire et al., 2004) were set up and maintained at 18°C. Adults were placed in new vials and transferred to 29°C for a week. To obtain the identical genetic backgrounds that are critical for aging experiments, control flies were generated for each experiment separately by outcrossing the GAL4 driver with the relevant control (Partridge & Gems, 2007). *miR‐34* null mutants obtained from Bonini's lab (Liu et al., 2012) were outcrossed for five generations with control flies (*w1118*), followed by crossing with a balancer for the 3rd chromosome (Sb/TM6b), yielding homozygotes *miR‐34* null. The new *miR‐34* null line was verified by PCR for *miR‐34* region deletion and for expression of the *miR‐277/dFMR1* rescue construct.

### 4.2 Generation of DNA constructs

Control *Ubi‐GFP* sensor and *Ubi‐miR‐34*‐sensor were generated in our laboratory by cloning the 3′UTR into the pKF254pUASTattB plasmid (a gift from K. Forstemann (Esslinger et al., 2013)). To generate the *Ubi‐miR‐34*‐sensor, the pKF254pUASTattB plasmid was digested with AvrII and NotI restriction enzymes and ligated with an 82 bp insert containing three repeats of the *miR‐34* antisense sequence (separated with a 3 bp spacer: CAG), at the 3’UTR after the GFP coding region. The insert was performed by annealing forward and reverse primers. Primers were re‐suspended to a concentration of 100 μM, and 5 μL of each diluted primer was added to a fresh tube, held 95°C for 4 min, and then gradually cooled to 24°C to allow annealing. The DNA constructs described above were verified by DNA sequencing. The *Ubi‐miR‐34*‐sensor plasmid, along with *Ubi‐GFP* sensor with no insert, were injected into *Drosophila* embryos by BestGene to generate transgenic lines.

### 4.3 Immunofluorescence

Whole‐mount testes from adult *Drosophila* were dissected in PBS and placed in Terasaki plates in 10 μL fix solution containing 2% paraformaldehyde in PBS for 1 h at RT, rinsed and washed twice in PBST (0.5% Triton X‐100 in PBS), followed by standard immunofluorescence staining. Samples were mounted in Vectashield mounting medium. Images were taken on a Nikon A1R confocal microscope or a Zeiss Axio Observer microscope equipped with an Apotome system using the AxioVison software and processed with Adobe Photoshop CS6.

### 4.4 RNA extraction

Testes of 100 flies (~200 testis) of each phenotype (i.e., young and aged) were dissected in PBS DEPC. Testes were collected and pooled in 100 μL TRIzol reagent and stored at −80°C until future RNA extraction. To maximize RNA extraction, frozen samples were thawed at 37°C and refrozen in liquid nitrogen (−80°C) five times, followed by 5 cycles of 30 s long cycles of vortexing and rest. Then, 100 μL of 99% ethanol were added and total RNA was extracted using a Direct‐zol RNA miniprep kit with DNAse treatment, according to the manufacturer's instructions. RNA was eluted in 50 μL of pre‐heated DNAse‐ and RNAse‐free water and kept at −80°C for future use. RNA quality was measured by a bioanalyzer and aliquots were used for transcriptome and qRT‐PCR analysis.

### 4.5 Transcriptome analysis

Illumina cDNA libraries were prepared from 1 μg total RNA extracted from testes of young and aged control *w1118* and *miR‐34* null mutants. Sequencing libraries were prepared using INCPM mRNA Sequence Single‐Read. Sixty reads were sequenced on two lanes of an Illumina HiSeq apparatus. The output was ~22 million reads per sample. Poly‐A/T stretches and Illumina adapters were trimmed from the reads using cutadapt. Resulting reads shorter than 30 bp were discarded. Reads were mapped to the *Drosophila melanogaster* dmel reference genome using STAR, supplied with gene annotations downloaded from FlyBase (r6.18) (and with the EndToEnd option and outFilterMismatchNoverLmax was set to 0.04). Expression levels for each gene were quantified using htseq‐count, using the gtf above. Differentially expressed genes were identified and analysis was performed using DESeq2 with the betaPrior, cooksCutoff and independent filtering parameters set to False. Raw P values were adjusted for multiple testing using the procedure of Benjamini and Hochberg. Differential expression data was filtered based on log fold change (logFC ≥ 0.8), and significance cutoff (*p* value ≤0.05) and minimal reading levels (CPM ≥ 1). The group of genes that showed higher expression in the *miR‐34* mutant versus controls in both young and aged testis was compared to a list of in silico‐predicted *miR‐34* targets (Targetscan Fly, http://www.targetscan.org/) (Ruby et al., 2007).

### 4.6 Western blotting

Testes of 100 young and aged flies (~200 testis) of each phenotype young and aged were dissected in cytoplasmic extraction buffer (CEB) with halt protease inhibitor from the subcellular protein fractionation kit for tissues. Fractionation of membranes and cytoplasm compartments was carried out according to the manufacturer's instructions. Proteins were separated on 10% SDS–PAGE gels, followed by Western blotting according to standard procedures. Nitrocellulose membranes containing cell protein lysates were incubated with primary and secondary HRP‐conjugated antibodies. Proteins were visualized by a Western blotting detection kit for horseradish peroxidase and quantified using a CCD camera and Image J software.

### 4.7 qRT–PCR

RNA (1 μg) was treated with DNaseI (Promega) and reverse‐transcribed with a random hexamer mixture and a High‐Capacity cDNA Reverse Transcription (RT) Kit (ThermoFischer Scientific) according to the manufacturer's instructions. Quantitative real‐time PCR was performed with a StepOnePlus Real‐time PCR System using SYBR Green PCR Master mix (Applied Biosystems). The efficiencies of target and reference amplification were approximately equal. Specific *αPS2* and βPS primers for qRT‐PCR were used. Levels were compared to the average of two normalizing genes, *act24A* and *sdhA*. For miRNA analysis, 10 ng RNA was used to prepare cDNA with TaqMan miRNA‐specific reverse‐transcription primers (Applied Biosystems) for *miR‐34* and ribosomal 2S RNA. Real‐time PCR results were analyzed using StepOne software (Applied Biosystems) and significance was determined using Student's *t*‐test. An average of three experiments (each performed in triplicate measurements) is shown (mean ± s.d.). *P* values were generated after a two‐tailed Student's *t*‐test was used to compare Δ*C*_T_ between time points or genotypes across three independent biological replicates.

### 4.8 Quantitative and statistical analysis

The volume germ cell debris was calculated from 10 Z stacks (1 μm each, above and beneath the hub) as LysoTracker‐positive cells. Quantification of gem cell debris volumes was performed using Imaris (Bitplane) software with an appropriate iso‐surfacing threshold. To determine statistical significance, Prism GraphPad version 8 software was used. First, normality and log normality were assessed using the Shapiro–Wilk test. In all experiments, there was no normal distribution, as expected from the different sizes of 2–16 dying interconnected spermatogonia. Therefore, nonparametric tests were conducted. An average of all experiments is shown as the mean and SD ± 95% confidence interval and the number (*n*) of testes examined. P values were generated using two‐tailed Mann–Whitney or Kruskal‐Wallis tests (depending on the number of samples) to compare time points or genotypes.

## AUTHOR CONTRIBUTIONS

Hila Toledano conceptualized and supervised the project, interpreted the data and wrote the manuscript. Noam Perry, Racheli Braun, Aya Ben‐Hamo‐Arad, Diana Kanaan, Tal Arad and Lilach Porat‐Kuperstein performed the experiments, collected data, performed analyses and review the manuscript.

## FUNDING INFORMATION

This work was supported by the Israel Science Foundation (ISF) personal grants (503/17 and 207/20) and by the United States‐Israel Binational Science Foundation (BSF) grant (2015398).

## CONFLICT OF INTEREST STATEMENT

The authors declare no competing interests.

## Supporting information


Movie S1.

Movie S2.

Data S1.

## Data Availability

The published transcriptome dataset is available at: https://doi.org/10.5061/dryad.tdz08kq58. All data are available in the main text or the supporting information. All reagents used in the study will be publicly available upon acceptance of the manuscript. The data that support the findings of this study are available from the corresponding author upon request.

## References

[acel14131-bib-0001] Agostini, M. , & Knight, R. A. (2014). miR‐34: From bench to bedside. Oncotarget, 5(4), 872–881. 10.18632/oncotarget.1825 24657911 PMC4011589

[acel14131-bib-0002] Alenzi, F. Q. , Alenazi, B. Q. , Ahmad, S. Y. , Salem, M. L. , Al‐Jabri, A. A. , & Wyse, R. K. (2009). The haemopoietic stem cell: Between apoptosis and self renewal. The Yale Journal of Biology and Medicine, 82(1), 7–18. https://www.ncbi.nlm.nih.gov/pubmed/19325941 19325941 PMC2660591

[acel14131-bib-0003] Allan, D. J. , Harmon, B. V. , & Roberts, S. A. (1992). Spermatogonial apoptosis has three morphologically recognizable phases and shows no circadian rhythm during normal spermatogenesis in the rat. Cell Proliferation, 25(3), 241–250. 10.1111/j.1365-2184.1992.tb01399.x 1596537

[acel14131-bib-0004] Boada‐Romero, E. , Martinez, J. , Heckmann, B. L. , & Green, D. R. (2020). The clearance of dead cells by efferocytosis. Nature Reviews. Molecular Cell Biology, 21(7), 398–414. 10.1038/s41580-020-0232-1 32251387 PMC7392086

[acel14131-bib-0005] Bokel, C. , & Brown, N. H. (2002). Integrins in development: Moving on, responding to, and sticking to the extracellular matrix. Developmental Cell, 3(3), 311–321. 10.1016/s1534-5807(02)00265-4 12361595

[acel14131-bib-0006] Boyle, M. , Wong, C. , Rocha, M. , & Jones, D. L. (2007). Decline in self‐renewal factors contributes to aging of the stem cell niche in the drosophila testis. Cell Stem Cell, 1(4), 470–478. 10.1016/j.stem.2007.08.002 18371382

[acel14131-bib-0007] Brennecke, J. , Hipfner, D. R. , Stark, A. , Russell, R. B. , & Cohen, S. M. (2003). Bantam encodes a developmentally regulated microRNA that controls cell proliferation and regulates the proapoptotic gene hid in drosophila. Cell, 113(1), 25–36. http://www.ncbi.nlm.nih.gov/pubmed/12679032 12679032 10.1016/s0092-8674(03)00231-9

[acel14131-bib-1000] Brown, N. H. , Gregory, S. L. , & Martin‐Bermudo, M. D. (2000). Integrins as mediators of morphogenesis in Drosophila. Developmental Biology, 223(1), 1–16. 10.1006/dbio.2000.9711 10864456

[acel14131-bib-0008] Burke, S. L. , Hammell, M. , & Ambros, V. (2015). Robust distal tip cell pathfinding in the face of temperature stress is ensured by two conserved microRNAS in Caenorhabditis elegans. Genetics, 200(4), 1201–1218. 10.1534/genetics.115.179184 26078280 PMC4574240

[acel14131-bib-0009] Cheng, J. , Turkel, N. , Hemati, N. , Fuller, M. T. , Hunt, A. J. , & Yamashita, Y. M. (2008). Centrosome misorientation reduces stem cell division during ageing. Nature, 456(7222), 599–604. 10.1038/nature07386 18923395 PMC2712891

[acel14131-bib-0010] Djuranovic, S. , Nahvi, A. , & Green, R. (2012). miRNA‐mediated gene silencing by translational repression followed by mRNA deadenylation and decay. Science, 336(6078), 237–240. 10.1126/science.1215691 22499947 PMC3971879

[acel14131-bib-0011] Epstein, Y. , Perry, N. , Volin, M. , Zohar‐Fux, M. , Braun, R. , Porat‐Kuperstein, L. , & Toledano, H. (2017). miR‐9a modulates maintenance and ageing of drosophila germline stem cells by limiting N‐cadherin expression. Nature Communications, 8(1), 600. 10.1038/s41467-017-00485-9 PMC5605507 28928361

[acel14131-bib-0012] Esslinger, S. M. , Schwalb, B. , Helfer, S. , Michalik, K. M. , Witte, H. , Maier, K. C. , Martin, D. , Michalke, B. , Tresch, A. , Cramer, P. , & Forstemann, K. (2013). Drosophila miR‐277 controls branched‐chain amino acid catabolism and affects lifespan. RNA Biology, 10(6), 1042–1056. 10.4161/rna.24810 23669073 PMC3904584

[acel14131-bib-0048] Garg, D. , & Cohen, S. M. (2014). miRNAs and aging: A genetic perspective. Ageing Research Reviews, 17, 3–8. 10.1016/j.arr.2014.04.001 24755363

[acel14131-bib-0013] Hanayama, R. , Tanaka, M. , Miwa, K. , Shinohara, A. , Iwamatsu, A. , & Nagata, S. (2002). Identification of a factor that links apoptotic cells to phagocytes. Nature, 417(6885), 182–187. 10.1038/417182a 12000961

[acel14131-bib-0014] He, L. , He, X. , Lim, L. P. , de Stanchina, E. , Xuan, Z. , Liang, Y. , Xue, W. , Zender, L. , Magnus, J. , Ridzon, D. , Jackson, A. L. , Linsley, P. S. , Chen, C. , Lowe, S. W. , Cleary, M. A. , & Hannon, G. J. (2007). A microRNA component of the p53 tumour suppressor network. Nature, 447(7148), 1130–1134. 10.1038/nature05939 17554337 PMC4590999

[acel14131-bib-0015] Hornik, T. C. , Vilalta, A. , & Brown, G. C. (2016). Activated microglia cause reversible apoptosis of pheochromocytoma cells, inducing their cell death by phagocytosis. Journal of Cell Science, 129(1), 65–79. 10.1242/jcs.174631 26567213 PMC4732292

[acel14131-bib-0016] Inaba, M. , Yuan, H. , & Yamashita, Y. M. (2011). String (Cdc25) regulates stem cell maintenance, proliferation and aging in drosophila testis. Development, 138(23), 5079–5086. 10.1242/dev.072579 22031544 PMC3210491

[acel14131-bib-0017] Issigonis, M. , Tulina, N. , de Cuevas, M. , Brawley, C. , Sandler, L. , & Matunis, E. (2009). JAK‐STAT signal inhibition regulates competition in the drosophila testis stem cell niche. Science, 326(5949), 153–156. 10.1126/science.1176817 19797664 PMC3073347

[acel14131-bib-0018] Janostiak, R. , Pataki, A. C. , Brabek, J. , & Rosel, D. (2014). Mechanosensors in integrin signaling: The emerging role of p130Cas. European Journal of Cell Biology, 93(10–12), 445–454. 10.1016/j.ejcb.2014.07.002 25062607

[acel14131-bib-0019] Kanaan, D. , Shklyar, B. , Porat‐Kuperstein, L. , & Toledano, H. (2023). Live imaging of Phagoptosis in ex vivo drosophila testis. Bio‐Protocol, 13(6), e4637. 10.21769/BioProtoc.4637 36968443 PMC10031521

[acel14131-bib-0020] Kennerdell, J. R. , Liu, N. , & Bonini, N. M. (2018). MiR‐34 inhibits polycomb repressive complex 2 to modulate chaperone expression and promote healthy brain aging. Nature Communications, 9(1), 4188. 10.1038/s41467-018-06592-5 PMC6180074 30305625

[acel14131-bib-0021] Lau, N. C. , Lim, L. P. , Weinstein, E. G. , & Bartel, D. P. (2001). An abundant class of tiny RNAs with probable regulatory roles in Caenorhabditis elegans. Science, 294(5543), 858–862. 10.1126/science.1065062 11679671

[acel14131-bib-0022] Liu, N. , Landreh, M. , Cao, K. , Abe, M. , Hendriks, G. J. , Kennerdell, J. R. , Zhu, Y. , Wang, L. S. , & Bonini, N. M. (2012). The microRNA miR‐34 modulates ageing and neurodegeneration in drosophila. Nature, 482(7386), 519–523. 10.1038/nature10810 22343898 PMC3326599

[acel14131-bib-0023] Lu, K. L. , & Yamashita, Y. M. (2017). Germ cell connectivity enhances cell death in response to DNA damage in the drosophila testis. eLife, 6, 1–16. 10.7554/eLife.27960 PMC5577909 28809158

[acel14131-bib-0024] Lu, Z. , Elliott, M. R. , Chen, Y. , Walsh, J. T. , Klibanov, A. L. , Ravichandran, K. S. , & Kipnis, J. (2011). Phagocytic activity of neuronal progenitors regulates adult neurogenesis. Nature Cell Biology, 13(9), 1076–1083. 10.1038/ncb2299 21804544 PMC3374401

[acel14131-bib-0025] Maartens, A. P. , & Brown, N. H. (2015). Anchors and signals: The diverse roles of integrins in development. Current Topics in Developmental Biology, 112, 233–272. 10.1016/bs.ctdb.2014.11.020 25733142

[acel14131-bib-0026] McGuire, S. E. , Mao, Z. , & Davis, R. L. (2004). Spatiotemporal gene expression targeting with the TARGET and gene‐switch systems in drosophila. Science's STKE, 2004(220), pl6. 10.1126/stke.2202004pl6 14970377

[acel14131-bib-0027] Morin, X. , Daneman, R. , Zavortink, M. , & Chia, W. (2001). A protein trap strategy to detect GFP‐tagged proteins expressed from their endogenous loci in drosophila. Proceedings of the National Academy of Sciences of the United States of America, 98(26), 15050–15055. 10.1073/pnas.261408198 11742088 PMC64981

[acel14131-bib-0028] Partridge, L. , & Gems, D. (2007). Benchmarks for ageing studies. Nature, 450(7167), 165–167. 10.1038/450165a 17994065

[acel14131-bib-0029] Penberthy, K. K. , & Ravichandran, K. S. (2016). Apoptotic cell recognition receptors and scavenger receptors. Immunological Reviews, 269(1), 44–59. 10.1111/imr.12376 26683144 PMC4685734

[acel14131-bib-0030] Perry, N. , Volin, M. , & Toledano, H. (2017). microRNAs in drosophila regulate cell fate by repressing single mRNA targets. The International Journal of Developmental Biology, 61(3–4‐5), 165–170. 10.1387/ijdb.160271ht 28621414

[acel14131-bib-0031] Rodriguez, I. , Ody, C. , Araki, K. , Garcia, I. , & Vassalli, P. (1997). An early and massive wave of germinal cell apoptosis is required for the development of functional spermatogenesis. The EMBO Journal, 16(9), 2262–2270. 10.1093/emboj/16.9.2262 9171341 PMC1169828

[acel14131-bib-0032] Ruby, J. G. , Stark, A. , Johnston, W. K. , Kellis, M. , Bartel, D. P. , & Lai, E. C. (2007). Evolution, biogenesis, expression, and target predictions of a substantially expanded set of drosophila microRNAs. Genome Research, 17(12), 1850–1864. 10.1101/gr.6597907 17989254 PMC2099593

[acel14131-bib-0033] Sapar, M. L. , Ji, H. , Wang, B. , Poe, A. R. , Dubey, K. , Ren, X. , Ni, J. Q. , & Han, C. (2018). Phosphatidylserine externalization results from and causes neurite degeneration in drosophila. Cell Reports, 24(9), 2273–2286. 10.1016/j.celrep.2018.07.095 30157423 PMC6174084

[acel14131-bib-0034] Serizier, S. B. , & McCall, K. (2017). Scrambled eggs: Apoptotic cell clearance by non‐professional phagocytes in the drosophila ovary. Frontiers in Immunology, 8, 1642. 10.3389/fimmu.2017.01642 29238344 PMC5712531

[acel14131-bib-0035] Sierra, A. , Encinas, J. M. , Deudero, J. J. , Chancey, J. H. , Enikolopov, G. , Overstreet‐Wadiche, L. S. , Tsirka, S. E. , & Maletic‐Savatic, M. (2010). Microglia shape adult hippocampal neurogenesis through apoptosis‐coupled phagocytosis. Cell Stem Cell, 7(4), 483–495. 10.1016/j.stem.2010.08.014 20887954 PMC4008496

[acel14131-bib-0036] Tanentzapf, G. , Devenport, D. , Godt, D. , & Brown, N. H. (2007). Integrin‐dependent anchoring of a stem‐cell niche. Nature Cell Biology, 9(12), 1413–1418. 10.1038/ncb1660 17982446 PMC3529653

[acel14131-bib-0037] Toledano, H. , D'Alterio, C. , Czech, B. , Levine, E. , & Jones, D. L. (2012). The let‐7‐imp axis regulates ageing of the drosophila testis stem‐cell niche. Nature, 485(7400), 605–610. 10.1038/nature11061 22660319 PMC4829122

[acel14131-bib-0038] Trappmann, B. , Gautrot, J. E. , Connelly, J. T. , Strange, D. G. , Li, Y. , Oyen, M. L. , Cohen Stuart, M. A. , Boehm, H. , Li, B. , Vogel, V. , Spatz, J. P. , Watt, F. M. , & Huck, W. T. (2012). Extracellular‐matrix tethering regulates stem‐cell fate. Nature Materials, 11(7), 642–649. 10.1038/nmat3339 22635042

[acel14131-bib-0039] Tung, T. T. , Nagaosa, K. , Fujita, Y. , Kita, A. , Mori, H. , Okada, R. , Nonaka, S. , & Nakanishi, Y. (2013). Phosphatidylserine recognition and induction of apoptotic cell clearance by drosophila engulfment receptor Draper. Journal of Biochemistry, 153(5), 483–491. 10.1093/jb/mvt014 23420848

[acel14131-bib-0040] Volin, M. , Zohar‐Fux, M. , Gonen, O. , Porat‐Kuperstein, L. , & Toledano, H. (2018). microRNAs selectively protect hub cells of the germline stem cell niche from apoptosis. The Journal of Cell Biology, 217(11), 3829–3838. 10.1083/jcb.201711098 30093492 PMC6219711

[acel14131-bib-0041] Wallenfang, M. R. , Nayak, R. , & DiNardo, S. (2006). Dynamics of the male germline stem cell population during aging of Drosophila melanogaster. Aging Cell, 5(4), 297–304. 10.1111/j.1474-9726.2006.00221.x 16800845

[acel14131-bib-0042] Wolfenson, H. , Lavelin, I. , & Geiger, B. (2013). Dynamic regulation of the structure and functions of integrin adhesions. Developmental Cell, 24(5), 447–458. 10.1016/j.devcel.2013.02.012 23484852 PMC3878073

[acel14131-bib-0043] Yacobi‐Sharon, K. , Namdar, Y. , & Arama, E. (2013). Alternative germ cell death pathway in drosophila involves HtrA2/Omi, lysosomes, and a caspase‐9 counterpart. Developmental Cell, 25(1), 29–42. 10.1016/j.devcel.2013.02.002 23523076

[acel14131-bib-0044] Yang, J. , Chen, D. , He, Y. , Melendez, A. , Feng, Z. , Hong, Q. , Bai, X. , Li, Q. , Cai, G. , Wang, J. , & Chen, X. (2013). MiR‐34 modulates Caenorhabditis elegans lifespan via repressing the autophagy gene atg9. Age (Dordrecht, Netherlands), 35(1), 11–22. 10.1007/s11357-011-9324-3 22081425 PMC3543738

[acel14131-bib-0045] Zaidel‐Bar, R. , Itzkovitz, S. , Ma'ayan, A. , Iyengar, R. , & Geiger, B. (2007). Functional atlas of the integrin adhesome. Nature Cell Biology, 9(8), 858–867. 10.1038/ncb0807-858 17671451 PMC2735470

[acel14131-bib-0046] Zervas, C. G. , Psarra, E. , Williams, V. , Solomon, E. , Vakaloglou, K. M. , & Brown, N. H. (2011). A central multifunctional role of integrin‐linked kinase at muscle attachment sites. Journal of Cell Science, 124(Pt 8), 1316–1327. 10.1242/jcs.081422 21444757 PMC3065386

[acel14131-bib-0047] Zohar‐Fux, M. , Ben‐Hamo‐Arad, A. , Arad, T. , Volin, M. , Shklyar, B. , Hakim‐Mishnaevski, K. , & Toledano, H. (2022). The phagocytic cyst cells in drosophila testis eliminate germ cell progenitors via phagoptosis. Science Advances, 8(24), eabm4937. 10.1126/sciadv.abm4937 35714186 PMC9205596

