# Supplementary material for: Integrin restriction by miR‐34 protects germline progenitors from cell death during aging
Source: Aging Cell. 2024 Mar 7;23(6):e14131. doi: 10.1111/acel.14131 (PMC11166360; doi:10.1111/acel.14131)
Supplement: Supplementary file 3 — Data S1. [file ACEL-23-e14131-s001.docx]

##
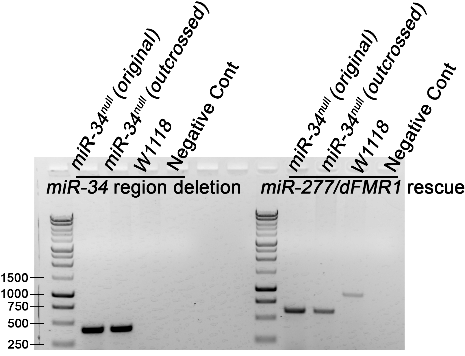
SUPPORTING INFORMATION

## Figure S1 Characterization of outcrossed *miR-34* null flies. *miR-34* null mutants obtained from Bonini's lab (Liu et al., 2012) were outcrossed for five generations with control flies (*W1118*), followed by crossing with a balancer for the 3^rd^ chromosome (Sb/TM6B), yielding the homozygous *miR-34* null mutants used in this research. A DNA gel showing PCR fragments of genomic DNA from original and outcrossed *miR-34 null* and *w1118* lines. *miR-34* deletion and expression of the *miR-277/dFMR1* rescue construct are shown for the original and outcrossed *miR-34 null* mutant.

##
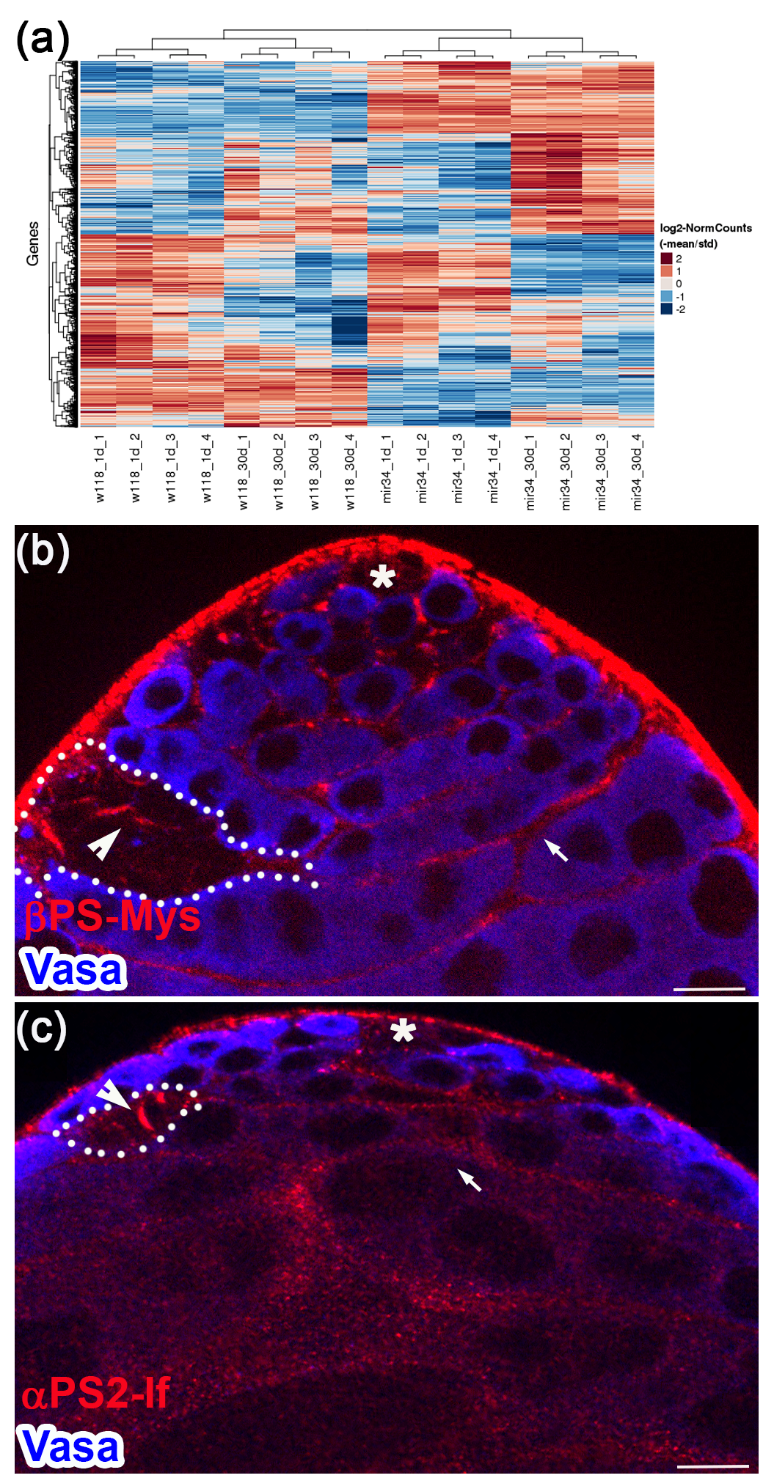


## Figure S2 Identification of *miR-34* mRNA targets. (a) Heatmap clustering of transcriptome analysis. Differentially expressed *Drosophila* genes from testes of 1- and 30-day old control (*w1118*) and *miR-34* null mutants. The heatmap color values correspond to the z scores of RNA-Seq CPM values. Horizontal axis: clustering by samples; Vertical axis: clustering by genes, where each color strip represents a clade of similarly expressed genes. Note clustering of each of four biological repeats and age. (b-c) Wild-type testes (*w1118*) were immunostained for Vasa (blue) and βPS (B, red) or αPS2 (C, red). The white dashed line delineates GCD events that appear by empty areas (“holes”) in the Vasa-stained tissues. Arrowheads mark the expression of βPS and αPS2 on the membrane of cyst cells that penetrate into notches of degraded germ cells. Arrows mark the expression of βPS and αPS2 in cyst cells. Asterisks mark the hub and scale bars represent 10 µm.

**Figure S3** ILK-GFP is expressed in cyst cells. Testes of ILK-GFP were stained with DAPI (white, nuclei) and immunostained for Vasa (blue) and GFP (green). Yellow arrows mark expression of ILK in cyst cells. Yellow arrowheads mark the germ cells. Note that ILK is not expressed in germ cells. Asterisks mark the hub and scale bars represent 10 µm.


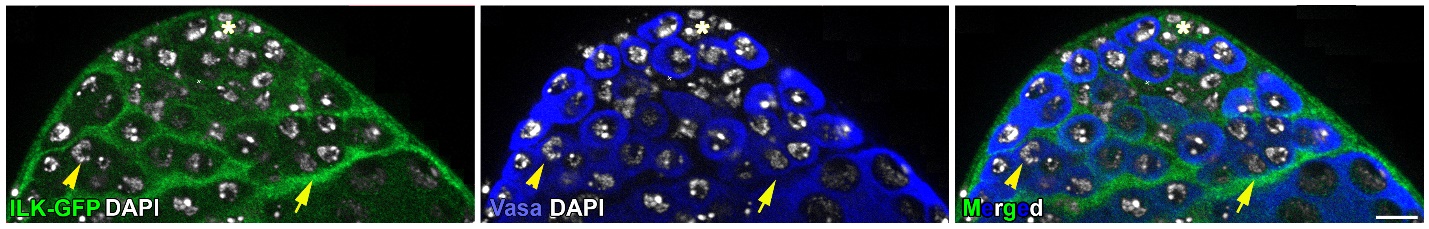


##
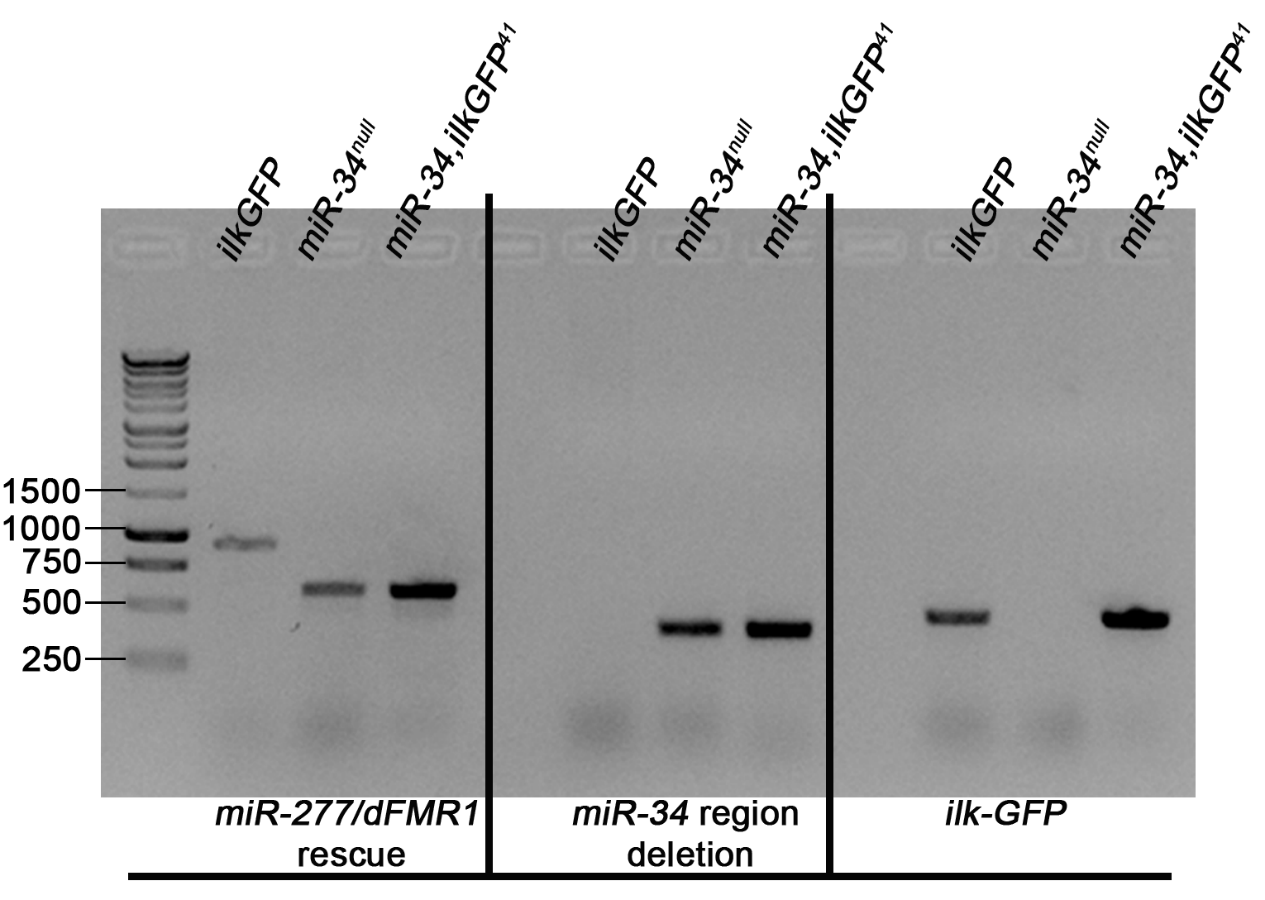


## Figure S4 Characterization of the *miR-34,IlkGFP^41^* recombinant line. A DNA gel showing PCR fragments of genomic DNA from transgenic lines of *ilk-GFP, miR-34 null* and *miR-34,ilkGFP^41^* recombinant flies. *miR-34* deletion and expression of the *miR-277/dFMR1* rescue construct are shown in *miR-34* and *miR-34,ilkGFP^41^* recombinant flies and absent from *ilk-GFP* flies (left and center). GFP is seen in *ilk-GFP* and *miR-34,ilkGFP^41^* and absent from *miR-34* null flies (right).

## Figure S5 Overexpression of integrin receptor subunits in cyst cells slightly demise the stem cell niche of aged males. Immunofluorescent images of testes from 30-day-old males of (a) control (*c587Gal4;UAS-cytGFP* outcrossed to *w1118*; n=31), (b) *βPS-*overexpressing (OE) (*c587Gal4;UAS-cytGFP/UAS-βps-GFP*; n=34) and (c) *βPS-* and *αPS2-*overexpressing flies (*c587Gal4;UAS-cytGFP/UAS-βps,UAS- αps2*; n=30). Note that only a few samples of *βPS-*overexpressing (b, 6%) and *βPS-* and *αPS2-*overexpressing (c, 13%) flies lost the stem cell niche, while most of the samples (94% of *βPS-*overexpressing and 87% of *βPS-* and *αPS2-*overexpressing flies contained a functional niche similar to control males. Asterisk marks the hub, yellow dots indicate GSCs attached to the hub and scale bars correspond to 10 μm.


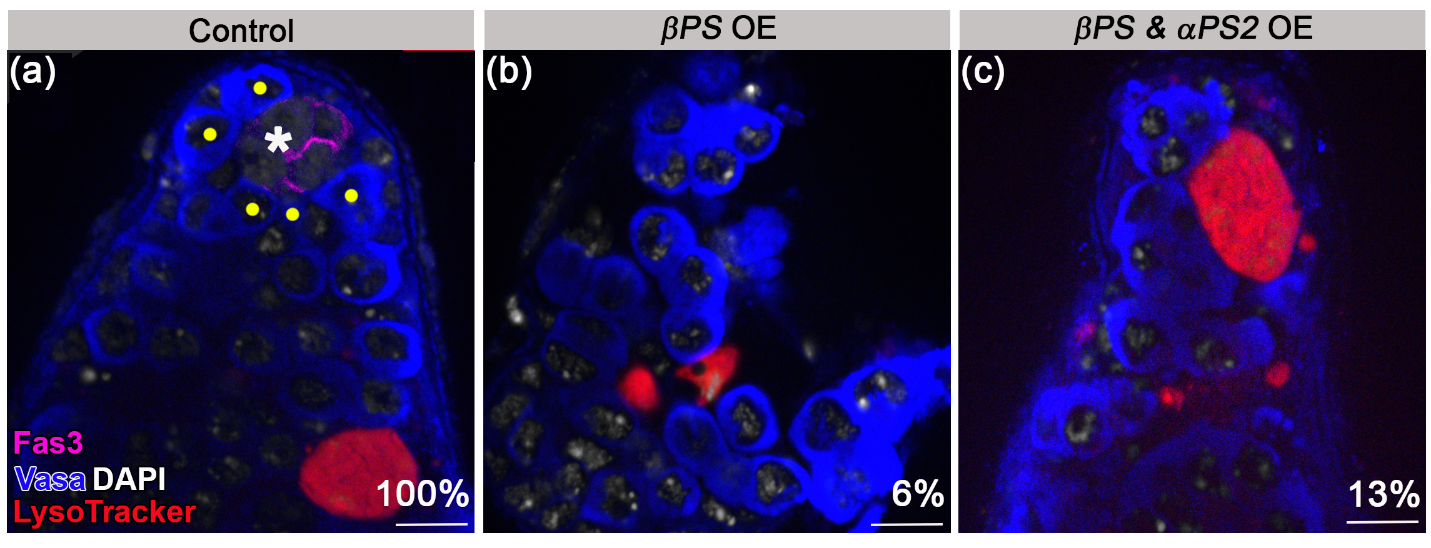


## Tables

|  |  |  | **Average reads** | |  |
| --- | --- | --- | --- | --- | --- |
| **Symbol** | **Log(FC)** | **P value (adj)** | ***w1118*** | ***miR-34*** | **seeds** |
| **cysu** | 4.8 | 7.5E-22 | 3.3 | 90.6 | 1 |
| **dysc** | 3.2 | 1.2E-20 | 9 | 82.6 | 1 |
| **uif** | 1.2 | 0.00015 | 45 | 180.2 | 1 |
| CG32206 | 1.1 | 0.04 | 6.9 | 19.3 | 1 |
| RhoGAP71E | 1.1 | 3.1E-6 | 1261.6 | 2720.9 | 1 |
| Mid1 | 1 | 0.0013 | 48.6 | 393.4 | 1 |
| **Cpr97Eb** | 1 | 9.25E-06 | 66.7 | 132 | 1 |
| CG13604 | 0.8 | 5.29E-07 | 154.1 | 271.7 | 1 |

## Table S1 Potential *miR-34* targets in the testis of young males. The table presents the eight genes increased in levels in testis from young *miR-34* mutants, relative to age-matched controls upon transcriptome analysis, relative to computationally predicted *miR-34* targets (<http://www.targetscan.org/>). Genes highlighted in bold were also increased in aged mutants and are presented in Appendix Table S2.

|  |  |  | **Average reads** | |  |
| --- | --- | --- | --- | --- | --- |
| **Symbol** | **Log(FC)** | **P value (adj)** | ***w^1118^*** | ***miR-34*** | **seeds** |
| **cysu** | 6.0 | 1.3E-13 | 1 | 67.5 | 1 |
| **dysc** | 3.1 | 1.7E-20 | 9 | 74.4 | 1 |
| swim | 2.4 | 1.1E-70 | 811.7 | 4287.3 | 2 |
| Eip74EF | 1.8 | 2.9E-14 | 122.1 | 429.1 | 2 |
| Thor | 1.8 | 8.8E-5 | 112.8 | 3785 | 1 |
| **Cpr97Eb** | 1.7 | 3.7E-13 | 31 | 101.7 | 1 |
| Clect27 | 1.5 | 0.02 | 13.7 | 39.5 | 1 |
| LanB2 | 1.5 | 1.4E-27 | 454.1 | 1312.6 | 1 |
| W | 1.2 | 1.5E-36 | 212.2 | 485.7 | 1 |
| Kul | 1.2 | 1.7E-14 | 165.3 | 373.5 | 1 |
| qsm | 1 | 7.9E-5 | 39.6 | 78.9 | 1 |
| Drep2 | 1 | 0.02 | 31.8 | 62.5 | 1 |
| wb | 0.9 | 8.96E-11 | 218.9 | 414.4 | 1 |
| **if (αPS2)** | **0.9** | **6.27E-15** | **270.3** | **507.6** | **1** |
| **mys (βPS)** | **0.9** | **1.19E-16** | **1273.9** | **2375** | **1** |
| CG6329 | 0.9 | 0.02 | 38.9 | 71.7 | 1 |
| **uif** | 0.9 | 0.004 | 79.1 | 145.7 | 1 |
| CG17124 | 0.9 | 7.94E-08 | 496.5 | 905.1 | 1 |
| pot | 0.8 | 1.75E-07 | 196.9 | 349.1 | 1 |

## Table S2 Potential *miR-34* targets in the testis of aged males. The table contains the 19 genes whose levels were increased in testis from aged *miR-34* null mutants, relative to age-matched controls upon transcriptome analysis, as compared to computationally predicted *miR-34* targets (<http://www.targetscan.org/>). The table includes the previously validated *Eip47EF* (fourth raw) and two integrin subunits receptor (bold and underlined) genes. Genes highlighted in bold were also increased in young mutants and are presented in Appendix Table S1.

|  |  |  | **Average reads** | |  |
| --- | --- | --- | --- | --- | --- |
| **Symbol** | **Log(FC)** | **P value (adj)** | ***w^1118^*** | ***miR-34*** | **seeds** |
| **mew (αPS1)** | **1.02** | **1.5E-07** | **226.755** | **459.5975** | **0** |
| **If (αPS2)** | **0.9103** | **6.27E-15** | **270.2875** | **507.595** | **1** |
| **scb (αPS3)** | **0.7842** | **2.01E-15** | **1336.453** | **2301.305** | **1*** |
| ItgaPS4 (αPS4) | 0.7478 | 0.0108 | 27.29 | 45.915 | 1 |
| ItgaPS5 (αPS5) | -0.2542 | 0.689 | 11.7675 | 9.9225 | 0 |

## Table S3 *Drosophila* αPS integrin receptor subunits in the testis of aged males. The table contains the levels of five αPS integrin receptor subunits in testis from aged *miR-34* null mutants, relative to age-matched controls, upon transcriptome analysis. αPS4-5 are poorly expressed in the testis. αPS1-3 (highlighted in bold) show high levels in the testes and are significant increased in *miR-34* null flies. * indicates a poorly conserved site in the ORF (<https://www.targetscan.org/fly_52orfs/>).

**Movie legends**

## Movie S1 The βPS integrin receptor subunit is expressed in phagocytic cyst cells during germ cell degradation (related to Figure 3d). A horizontal (360˚) view of the apical tip of the testis. Testes were stained with LysoTracker (red, GCD events) and DAPI (white, all nuclei), and immunostained for *βPS* (green). Shown is a 3D projection of a confocal image (Z-stack of 10 slices, each 1 µm-thick). The movie begins with a surface reconstruction rendered with Imaris software and ends with the original image. Note that *βPS* is expressed at the membrane of cyst cells that penetrate into notches of degraded germ cells.

## Movie S2 ILK-GFP is expressed in all cyst cells and dynamically changes localization in phagocytic cyst cells during GCD events (related to Figure 4a-c). A representative example of the apical tip of live, *ex vivo* *Drosophila* testis labeled with LysoTracker (red) and Hoechst stain (blue, nuclei). Testes from young transgenic flies expressing a protein trap of ILK-GFP. Time (h:min) is shown on the bottom of the movie and single channels are presented in grayscale. ILK GFP (green) is expressed in all cyst cells and changes localization in GCD events. Arrow marks an ongoing GCD event that leads to complete degradation over 5 h. Arrowhead marks a second *de novo* GCD event begins after 50 min with acidification and packed DNA in separate nuclei that become involuted into a single bundle.
